# Multifunctional metal–organic frameworks as promising nanomaterials for antimicrobial strategies

**DOI:** 10.1093/burnst/tkaf008

**Published:** 2025-01-24

**Authors:** Qian-Jin Li, Fei Xing, Wen-Ting Wu, Man Zhe, Wen-Qian Zhang, Lu Qin, Li-Ping Huang, Long-Mei Zhao, Rui Wang, Ming-Hui Fan, Chen-Yu Zou, Wei-Qiang Duan, Jesse Li-Ling, Hui-Qi Xie

**Affiliations:** Department of Orthopedic Surgery and Orthopedic Research Institute, Stem Cell and Tissue Engineering Research Center, State Key Laboratory of Biotherapy, West China Hospital, Sichuan University, Chengdu, China; Department of Pediatric Surgery, Division of Orthopedic Surgery, Orthopedic Research Institute, Laboratory of Stem Cell and Tissue Engineering, State Key Laboratory of Biotherapy, West China School of Medicine, West China Hospital, Sichuan University, No. 37 Guoxue Lane, Chengdu 610041, China; Department of Pediatric Surgery, Division of Orthopedic Surgery, Orthopedic Research Institute, Laboratory of Stem Cell and Tissue Engineering, State Key Laboratory of Biotherapy, West China School of Medicine, West China Hospital, Sichuan University, No. 37 Guoxue Lane, Chengdu 610041, China; Animal Experiment Center, West China Hospital, Sichuan University, No. 37 Guoxue Lane, Chengdu 610041, Sichuan, China; Department of Orthopedic Surgery and Orthopedic Research Institute, Stem Cell and Tissue Engineering Research Center, State Key Laboratory of Biotherapy, West China Hospital, Sichuan University, Chengdu, China; Integrated Care Management Center, West China Hospital, Sichuan University, No. 37 Guoxue Lane, Chengdu 610041, Sichuan, China; Department of Orthopedic Surgery and Orthopedic Research Institute, Stem Cell and Tissue Engineering Research Center, State Key Laboratory of Biotherapy, West China Hospital, Sichuan University, Chengdu, China; Department of Orthopedic Surgery and Orthopedic Research Institute, Stem Cell and Tissue Engineering Research Center, State Key Laboratory of Biotherapy, West China Hospital, Sichuan University, Chengdu, China; Department of Orthopedic Surgery and Orthopedic Research Institute, Stem Cell and Tissue Engineering Research Center, State Key Laboratory of Biotherapy, West China Hospital, Sichuan University, Chengdu, China; Department of Orthopedic Surgery and Orthopedic Research Institute, Stem Cell and Tissue Engineering Research Center, State Key Laboratory of Biotherapy, West China Hospital, Sichuan University, Chengdu, China; Department of Orthopedic Surgery and Orthopedic Research Institute, Stem Cell and Tissue Engineering Research Center, State Key Laboratory of Biotherapy, West China Hospital, Sichuan University, Chengdu, China; Department of Plastic and Burn Surgery, West China Hospital, Sichuan University, No. 37 Guoxue Lane, Chengdu 610041, Sichuan, China; Department of Medical Genetics, West China Second Hospital, Sichuan University, Chengdu 610041, China; Tianfu Jincheng Laboratory, Chengdu, 610093, China; Department of Orthopedic Surgery and Orthopedic Research Institute, Stem Cell and Tissue Engineering Research Center, State Key Laboratory of Biotherapy, West China Hospital, Sichuan University, Chengdu, China; Tianfu Jincheng Laboratory, Chengdu, 610093, China

**Keywords:** Metal–organic frameworks, Antimicrobial, Antibacterial, Bacteria, Infection

## Abstract

Bacterial infections pose a serious threat to human health. While antibiotics have been effective in treating bacterial infectious diseases, antibiotic resistance significantly reduces their effectiveness. Therefore, it is crucial to develop new and effective antimicrobial strategies. Metal–organic frameworks (MOFs) have become ideal nanomaterials for various antimicrobial applications due to their crystalline porous structure, tunable size, good mechanical stability, large surface area, and chemical stability. Importantly, the performance of MOFs can be adjusted by changing the synthesis steps and conditions. Pure MOFs can release metal ions to modulate cellular behaviors and kill various microorganisms. Additionally, MOFs can act as carriers for delivering antimicrobial agents in a desired manner. Importantly, the performance of MOFs can be adjusted by changing the synthesis steps and conditions. Furthermore, certain types of MOFs can be combined with traditional photothermal or other physical stimuli to achieve broad-spectrum antimicrobial activity. Recently an increasing number of researchers have conducted many studies on applying various MOFs for diseases caused by bacterial infections. Based on this, we perform this study to report the current status of MOF-based antimicrobial strategy. In addition, we also discussed some challenges that MOFs currently face in biomedical applications, such as biocompatibility and controlled release capabilities. Although these challenges currently limit their widespread use, we believe that with further research and development, new MOFs with higher biocompatibility and targeting capabilities can provide diversified treatment strategies for various diseases caused by bacterial infections.

## Graphical Abstract

**Graphical Abstract:**
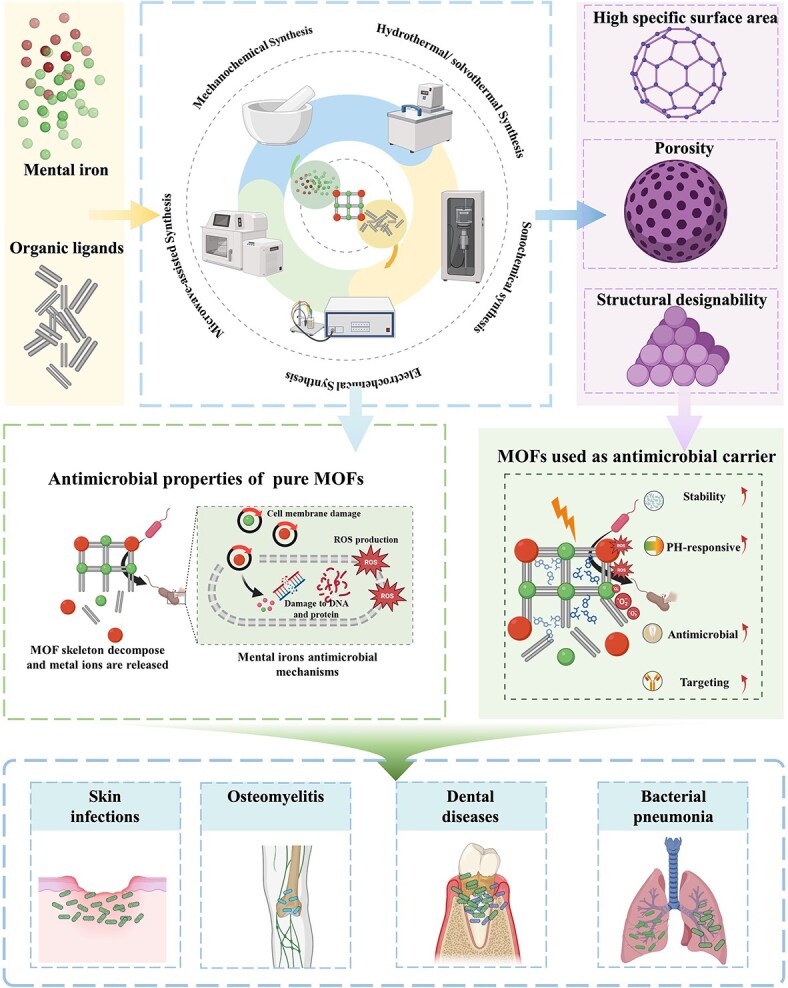

Highlights

- Metal–organic framework–based antimicrobial strategies are reviewed.
- The synthesis and antimicrobial properties of metal–organic frameworks used for antimicrobial applications are summarized.
- Metal–organic frameworks used as an antimicrobial carrier is overviewed.
- The current status and challenge of metal–organic frameworks for antimicrobial applications are prospected.

## Background

Bacterial infections are a serious threat to human health. Antibiotics provide a good strategy for the successful treatment of bacterial infectious diseases. The mortality and morbidity rates associated with various infectious diseases have significantly declined since the introduction of antibiotics [1]. However, with the widespread use of antibiotics and the continuous development of medical science, more and more difficulties in effectively controlling infectious diseases in the long term are being recognized. Antibiotic resistance (ABR) is one of the significant problems in these difficulties [2]. The threat of ABR has been well understood based on the study of the mechanism of bacterial resistance [3]. A series of measures have been taken to reduce the overuse of antibiotics to curb the development of ABR [3–6]. In addition, some physiological barriers, such as the blood–brain barrier, can serve as a protective mechanism to prevent the rapid entry of drugs, posing a challenge in effectively treating infections in the central nervous system [7]. All these factors contribute to the ineffectiveness of conventional antibiotic therapy in treating infectious diseases.

In recent years, nanomaterials have been widely studied in biomedicine due to their unique physiochemical properties and biological functions. The ability of many nanomaterials to destroy microbial cells has inspired new antimicrobial strategies [8, 9]. As a new type of porous nanomaterial under development, metal–organic frameworks (MOFs) have been studied in various fields such as energy development, water treatment, and microsensors [10–15]. Just like many other nanomaterials, the potential of MOFs for biomedical applications, including drug delivery, biosensing, bioimaging, tissue engineering, and regenerative medicine, has gained extensive attention [16]. MOFs are a class of organic–inorganic hybrid nanomaterials composed of polydentate organic ligands and metal ions or clusters through coordination bonds [17]. Porosity, high specific surface area, and structural designability are the most prominent features of MOFs, which are also the basis for their application in various fields [18, 19]. In addition, the structure design of MOFs can be easily achieved by modifying the raw materials and synthesis process. This means that the performance of MOFs, such as porosity and pore size, can be designed and adjusted according to specific application requirements [19–22].

With the growing trend of MOFs in the biomedical field, the development potential of MOFs in antimicrobials has gradually emerged. Just like many other classes of nanomaterials, MOFs have also been found to possess antibacterial properties [9, 23]. The antimicrobial activity of MOFs is mainly related to the metal components [24]. MOFs can exert a sustained antimicrobial effect by steadily and continuously releasing metal ions [24–27]. Positively charged metal ions can interact with negatively charged bacterial membranes to produce antimicrobial effects, and this antimicrobial activity is correlated with the concentration, surface area, particle size, and type of MOFs [25, 28]. The right particle size plays a crucial role in its uptake by cells and subsequent transport within the biological system. In addition to their antibacterial effect, MOFs have excellent drug-loading capacity due to their unique porous structure and high specific surface area [29, 30]. Using MOF loading with antimicrobial agents to produce synergistic antimicrobial effects, the effect of antimicrobial methods based on MOFs can be further improved while reducing the dose and the incidence of toxic side effects caused by MOFs and antimicrobial agents. In addition, MOFs that can adapt to different drug loads and have different drug release rates can be fabricated by changing precursors and synthetic conditions [29, 31]. Similarly, the controllable local release of antimicrobial drugs can be achieved by selecting the corresponding MOF carrier according to the local environmental pH of different microbial infections, thus reducing the systemic damage of antimicrobial drugs to the body. Some studies have also designed and produced drug carriers with core–shell nanostructures by the utilization of MOFs [32–34]. This design further enhances the functional diversity of MOFs, such as changing the stability of MOFs to control the rate of drug release [32] and endowing the MOFs with magnetism to enable drug locational release [33, 34]. MOF materials that respond to multiple stimuli and respond quickly and efficiently can precisely control drug release at specific times and locations and can be used to treat deep tissue infections with improved bioavailability and antimicrobial properties. Overall, these properties manifest MOFs’ great potential in antimicrobial therapy. It is reasonable to believe that developing antimicrobial strategies based on MOFs will significantly help address today’s challenges of anti-infective therapy. This review discussed the current status and unique challenges of using MOFs in antimicrobial treatment. Furthermore, the existing antibacterial mechanisms, the construction method of MOFs, and the specific antimicrobial application of MOFs were also discussed.

## Review

### The antimicrobial mechanism of metal–organic frameworks

With the rise of drug-resistant bacteria, there is an increasing focus on the use of MOFs for preventing bacterial attachment, inhibiting bacterial growth, and eradicating bacteria. MOFs are mixed inorganic–organic materials composed of metal ions and organic linkers. When the MOF skeleton decomposes, metal ions are released. Many metal ions such as silver ions (Ag^+^) and zinc ions (Zn^2+^) have been proven to have antibacterial activity. MOFs themselves possess antimicrobial abilities and can serve as antimicrobial agents. Their unique properties make them ideal for multifunctional applications, serving as carriers for various agents to achieve sustained release. Moreover, MOFs can be combined with other materials or synergized through multiple mechanisms to effectively combat bacteria. This review will delve into the detailed antimicrobial mechanisms of MOFs when used independently and as carriers.

**Figure 1:**
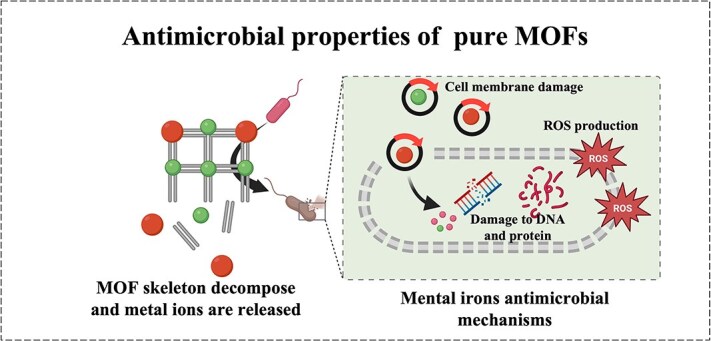
Antimicrobial properties of MOF. *MOF* metal–organic framework, *ROS* reactive oxygen species

The antibacterial ability of pure MOFs mostly comes from the metal ions in them. Many different metals have overlapping antimicrobial mechanisms due to similarities in physical and chemical properties [35]. Mainly through the following three antibacterial ways: positively charged metal ions have been shown to be able to attach to negatively charged bacterial membranes through electrostatic interactions, thereby destroying the bacterial membranes. In addition, MOFs can affect membrane function and integrity by interfering with membrane potential and damaging membrane structure [36]. For example, Ag^+^ was found to interfere with the function of membrane proteins, leading to membrane dysfunction and increased membrane permeability [37], while Cu^2+^ and Cd^2+^ are considered to cause membrane structure damage by mediating lipid peroxidation [38, 39]. Another crucial antimicrobial mechanism of metal ions is the production of reactive oxygen species (ROS) and the consumption of antioxidants [35]. For example, some redox-active metals such as Fe, Cu, Cr, and Co can catalyze the conversion of hydrogen peroxide into harmful hydroxyl radicals by mediating the Fenton reaction [40].Meanwhile, some thiophilic metals, such as Ag^+^, Pb^2+,^ and Cd^2+^, can consume the antioxidant reserves in bacterial cells by oxidizing glutathione (GSH), making cells vulnerable to ROS damage [40–42]. In addition to the above two antibacterial abilities, the damage of metal ions to bacterial cell proteins, enzymes, and DNA is also a proven antibacterial mechanism of metal-based substances [40]. For example, Ag^+^ has been shown to interact with DNA and render it unable to replicate [43]. Lots of metal ions such as Ag^+^, Cu^2+^, Cd^2+^, Hg^2+,^ and Zn^2+^ can target the thiol groups of many crucial proteins and thus affect the function of proteins and enzymes [44–46]. In short, through a series of molecular damage mechanisms, the normal biochemical reactions and metabolic activity cells of microorganisms are blocked, eventually leading to the death of microorganisms (Figure 1).

Benefitting from its high porosity and large surface area, an MOF possesses excellent molecular loading capacity, which lays a foundation for the construction of a drug loading system based on MOF [31]. However, in addition to the great drug-loading capability, the MOF also has a number of other special advantages in antimicrobial drug delivery. For example, the MOF itself can exert an antimicrobial effect, and its synergistic antimicrobial effect with many other antimicrobials has been demonstrated in several studies [47–49]. Moreover, the designability of structure and properties also enables the MOF to be adapted to various drug delivery requirements, and even complete some specialized targeted drug delivery work [50]. The antimicrobial mechanism of MOF loading with antibiotics and metal-based inorganics is described in detail below.

With the continuous development of medical science, people have a more and more thorough understanding of the antimicrobial mechanisms of antibiotics, while the emergence of new members of the antibiotic family is getting slower and slower [51]. However, although the development of antibiotics today is not as rapid as it was in the mid-20th century [52], the family of antibiotics is still very large. The targets of these various antibiotics cover the process of cell wall synthesis, protein translation, and DNA replication of microorganisms [53]. In addition, some antibiotics also exert antibacterial ability by acting on cell membranes and adenosine triphosphate (ATP) metabolism [6]. As the earliest found and most widely studied and used antibiotics, β-lactam antibiotics (e.g. penicillin, cephalosporin, carbapenem, oxacephems, and monobactams) play their role by influencing the synthesis of the peptidoglycan layer of bacterial cell wall [54]. The process of protein translation is another target of some antibiotics. Aminoglycosides, tetracyclines, macrolides, and oxazolidinones all exert antimicrobial effects mainly by inhibiting the synthesis of bacterial proteins [53]. In addition, interference with the normal synthesis and function of bacterial genetic material is also the way some antibiotics play their antibacterial role. Quinolones mainly act on DNA gyrase and topoisomerase IV to form a stable covalently linked drug-enzyme-DNA cleaved complex, which inhibits DNA supercoiling and relaxation processes, thus blocking DNA replication and transcription and ultimately leading to the death of bacteria [55]. In addition to the traditional antibiotic targets mentioned above, there are several kinds of antibiotics that can achieve antibacterial effects by affecting the properties and functions of cell membranes or hindering the metabolism of ATP in bacterial cells. The cyclic lipodepsipeptides daptomycin is supposed to be able to bind to the phosphatidyl glycerol in bacterial membranes and insert into the cell plasma membrane through a calcium-dependent process [56, 57].

While it may seem absurd that the family of antibiotics with such rich antibacterial mechanisms has not fully solved the problem of infectious diseases for us, there are many limitations to the practical use of antibiotics. Antibiotic resistance is one of the most famous ones. After a long struggle against antibiotics, today’s pathogenic microorganisms have evolved lots of methods to resist the action of antibiotics, including antibiotic destruction, antibiotic modification, and changing the target of antibiotics and antibiotic efflux pump [58]. However, as mentioned above, the emergence of new antibiotics is significantly slower than before, which suggests that we may need to incorporate the antibacterial mechanisms of other antibacterial substances to fight today’s pathogens. Of course, in addition to the notorious bacterial resistance that reduces the effectiveness of antibiotics, the toxicity of antibiotics and the direct administration strategy are also very important limiting factors [59]. Currently, due to the direct administration mode, the distribution, metabolic rate, and bioavailability of antibiotics *in vivo* are often difficult to regulate and directly dependent on the biochemistry of the antibiotic itself. Increasing the antibiotic dose and prolonging the administration time in order to strengthen the effect of antibiotics on the local infection site will increase the risk of toxic and side effects of antibiotics to the body, as well as cause double infections and inducing drug resistance [60, 61]. Therefore, combining antimicrobial mechanisms and improving drug delivery strategies may be important ways to bring about a turnaround in the current pessimistic situation of antibiotic use in addition to the development of new antibiotics. It is urgent to improve the antimicrobial efficiency of antibiotics without compromising or even reducing their usage. Loading antibiotics into MOFs with high porosity, controlled release behavior, and good biocompatibility is considered to be a very suitable solution. For instance, the effectiveness of amoxicillin and clavulanate potassium in treatment may be compromised by their limited ability to penetrate cell membranes. However, when loaded onto MOFs, they can enter cells through the process of phagocytosis to achieve intracellular antibacterial activity [62]. Loading onto MOFs can also address the shortcomings of vancomycin and other antibiotics that are unstable in water. Furthermore, different types of antibiotics and MOFs will result in different antimicrobial mechanisms, which will be elaborated upon with specific examples later.

While most metal-based inorganic antimicrobials are thought to exert antimicrobial effects primarily through the metal-related mechanisms described above, the same mechanism of action as pure MOFs, many novel inorganic antimicrobials, especially metal-based nanomaterials, exhibit other antimicrobial mechanisms due to their special properties. While most metal-based inorganic antimicrobials are perceived to exert antibacterial effects primarily through the metal-related mechanisms described above, many novel inorganic antimicrobials, especially metal-based nanomaterials, have been found to exhibit other antimicrobial mechanisms due to their special properties. For example, metal and metal oxide nanoparticles (NPs) can induce the formation of ROS through surface defect–based catalysis and electrochemical corrosion reactions [63]. Meanwhile, metal oxide nanoparticles such as TiO_2_ and ZnO_2_ can also produce ROS through a photocatalysis effect [9, 63]. Some nano-antibacterial materials can directly cause the destruction of bacterial cell membranes through mechanical damage. For example, MoS_2_ nanosheets, which can also induce ROS production through photocatalytic effect under light conditions, can penetrate or pierce the cell membrane with their sharp edges under dark conditions, causing local membrane damage and the leakage of intracellular components, ultimately leading to the bacterial cell death [64, 65]. In addition, some noble metal nanomaterials such as Au, Ag, and Pt, as well as some metallic compound nanomaterials such as MoS_2_, CuS, and MnO_2_, have been found to have photothermal antibacterial effects, which refers to the ability to generate heat by absorbing specific wavelengths light energy and thereby induce bacterial death [66]. Inorganic antibacterial agents have diverse ways of functioning compared to antibiotics, which suggests their potential for use in treating infections. However, these antibacterial agents lack specificity. Enhancing their characteristics and application strategies could overcome the limitations of inorganic antibacterial drugs. The MOF itself possesses antibacterial properties. The metal ions and organic ligands found in MOF materials have been demonstrated to have antibacterial effects. Additionally, MOFs have unique features such as high porosity, adjustable pore size, and large surface area, allowing them to encapsulate inorganic antibacterial agents within their pores. By creating a porous structure, sustained and controlled release can be achieved. The combination of inorganic antibacterial agents and the MOF can serve as a platform to maximize the antibacterial effect by leveraging multiple mechanisms.

**Figure 2:**
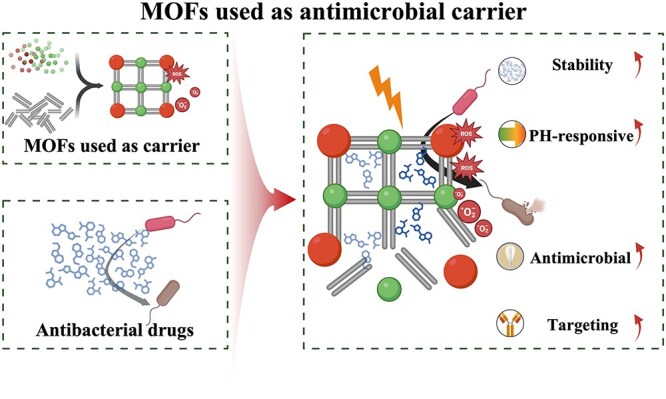
MOFs used as antimicrobial carrier. *MOF* metal–organic framework

It is frequently challenging to attain substantial sterilization effectiveness at low concentrations and over a shorter duration using a solitary antimicrobial mechanism. MOFs can serve as carriers for antibiotics, metal nanoparticles, metal oxide nanoparticles, etc., thereby enhancing the antibacterial properties of the loaded drugs and bolstering the stability of antimicrobial agents. Furthermore, not only can MOFs function as carriers but also, through appropriate manipulation, they can be engineered to shield drugs from external threats and facilitate controlled drug release (Figure 2).

### The synthesis of metal–organic frameworks

**Figure 3:**
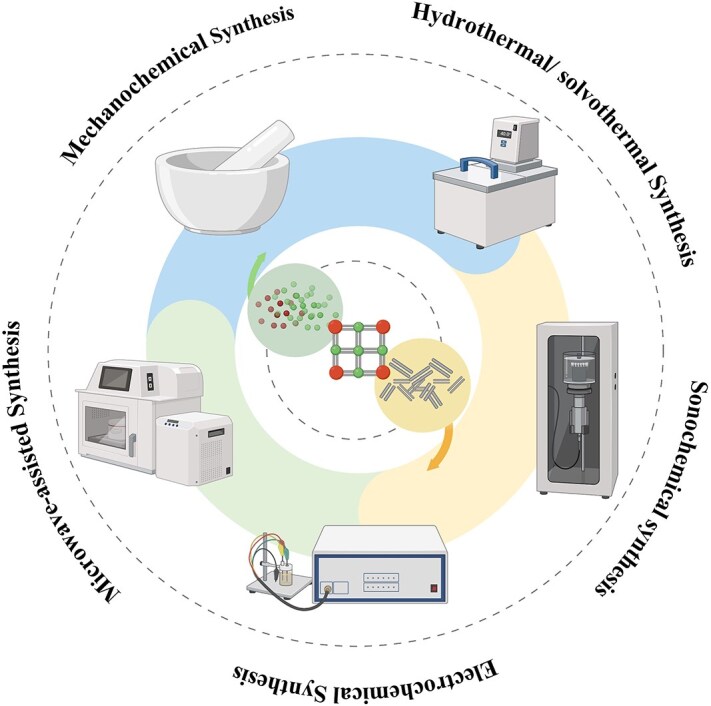
The construction method of MOF. *MOF* metal–organic framework

Synthesis of MOF with stable properties and specific structure is the premise of its application [67]. The change of synthesis routes and many factors in the synthesis process, such as ingredient proportion, solvent, temperature, and pH of reaction system, may lead to the obtained MOF with a completely different structure, particle size, and porosity, thus affecting the performance of the products [68]. Therefore, the understanding of MOF synthesis method is very important for obtaining the MOF that meets specific application requirements. As the earliest MOF synthesis method developed in 1995, solvothermal synthesis is still commonly used in laboratory construction of MOFs today [69, 70]. However, due to the limitations of solvothermal synthesis including low efficiency, high energy consumption, unsatisfactory product yield and quality, and difficult-to-achieve industrial-scale production, many alternative synthesis schemes have been explored and developed, such as electrochemical synthesis, microwave-assisted synthesis, sonochemical synthesis, mechanochemical synthesis, and other synthesis technologies [67, 69, 71], shown as Figure 3 in this section; we will briefly introduce these MOF synthesis techniques.

#### Hydrothermal/solvothermal synthesis

Hydrothermal/solvothermal reaction refers to the chemical reaction in a closed system where the temperature is above the boiling point of the water or nonwater solvent [72]. The process of solvothermal synthesis of MOF can be simply described as follows. First, the metal salt and the organic linker are dissolved in the solvent and then mixed or the metal salt, the organic linker, and the solvent are directly added together into the closed reaction vessel. Then, by heating to create a reaction environment of high temperature and pressure, the process of dissolution, interaction, and crystallization of the reactants is facilitated. Finally, insoluble MOF crystals grow and precipitate continuously on the container wall. Although the solvothermal synthesis of MOF seems not complicated, the properties and yield of MOF synthesized in this way are affected by a number of reaction parameters, including solvent system, reactant ratio, temperature, pressure, and reaction time [73]. Therefore, for the solvothermal method, it is necessary to carefully regulate the reaction conditions to get the desired MOF.

Currently, many researchers utilize hydrothermal/solvothermal synthesis to modulate the properties of MOFs. Li *et al*. tried to adjust the morphology and size of ZIF-78 crystals by changing the crystallization duration and the molar ratio of substrates to solvent during the solvothermal synthesis of ZIF-78 [74]. Chen *et al*. synthesized ZIF-8 with a hydrangea-like structure by the solvothermal method and observed that, with the increase of reaction temperature, the particle size of the obtained product gradually increased. Meanwhile, with the prolongation of reaction time, the crystal particle morphology gradually transformed into the classic hexagonal and rhomboidal polyhedron [75]. However, although we have known that the basic physical properties of MOF can be controlled by adjusting the solvothermal synthesis parameters, and the antibacterial properties of MOF are indeed related to the properties of MOF, there is still a lack of research on the exact relationship between the basic properties of MOF (such as particle size and morphology) and the properties directly related to antibacterial (such as the rate of metal ions release). Therefore, it remains to be further verified whether an MOF more suitable for antibacterial applications can be obtained by adjusting the synthesis conditions in solvothermal synthesis.

#### Microwave-assisted synthesis

In fact, the use of microwave energy signatures to heat reactants is not uncommon in synthetic chemistry, and it has shown some advantages in the synthesis of MOF. In the traditional solvothermal synthesis of MOF, the heat energy required by the reaction is usually provided by an external electric heating device such as an oven, and transferred to the internal reaction system through the closed container wall. However, in the microwave-assisted synthesis of MOF, the interaction between electromagnetic waves and polar molecules in the solution causes the dipole rotation of polar molecules so that the molecules quickly turn and try to orient themselves. Then, the friction and collision caused by the rapid movement of molecules generate the heat energy needed for the reaction, so as to realize more uniform and rapid heating of the reaction system. This microwave-assisted solvothermal synthesis allows direct heating of the reactants at a faster heating rate, and the crystallization of the product occurs not on the walls of the container, but at hot spots formed by heating inside the solution, thus significantly increasing the reaction efficiency. Jhung *et al*. achieved faster synthesis of chromium terephthalate MIL-101 by microwave-assisted synthesis (40 min at 210°C compared with 10 h at 220°C for conventional solvothermal synthesis) [76]. Gecgel *et al*. synthesized aluminum-based MOF MIL-53(Al) by microwave-assisted synthesis and studied the effects of MIL-53(Al) on various biological activities. They found that the synthesis of MIL-53(Al), which usually takes 3 days by the solvothermal method, can be completed in a water environment within 3 h by the microwave-assisted method, and the particle size of MIL-53(Al) synthesized by the microwave method is smaller than that synthesized by traditional solvothermal method. In addition, they observed and verified the antioxidant, DNA cleavage, antibacterial, and antibiofilm activities of the MIL-53(Al) obtained, suggesting that this aluminum-based MOF, which can be efficiently obtained through microwave-assisted route, has the potential to be used in antimicrobial therapy [77].

#### Sonochemical synthesis

Similar to the purpose of microwave-assisted synthesis, sonochemical synthesis is actually another way to provide the required energy for MOF synthesis, so it can also be called ultrasonic-assisted synthesis. In fact, ultrasonic waves do not interact directly with material molecules to produce thermal effects, as microwaves do. However, under the action of sufficiently high-energy ultrasonic waves, due to the uneven distribution of sound waves and the characteristics of superposition and attenuation, the acoustic compression phase and rarefaction phase are formed in the liquid medium affected by ultrasonic wave [78]. The liquid in the rarefaction phase will be subjected to tensile stress, which decreases the local pressure and forms bubbles. These bubbles constantly oscillate, grow, and accumulate energy under the action of ultrasonic field. When the energy and sound pressure reach the threshold, the bubbles will collapse sharply and rapidly release a large amount of energy. This process of the formation, growth, and collapse of bubbles inside the liquid under the effect of high-energy ultrasound is called cavitation, which can generate transient high temperature and high pressure (5000 K, 1000 bar) locally, thus driving various chemical reactions [79–81]. Therefore, rapid synthesis of MOF at room temperature can be achieved by utilizing the cavitation of ultrasonic waves.

In order to understand the specific beneficial effects of ultrasonic and microwave irradiation on MOF synthesis, Haque *et al*. designed experiments to compare the synthesis rates of Iron terephthalate MIL-53(Fe) under relatively low-temperature conditions of traditional electric heating, microwave assisted, and ultrasonic assisted. Their experimental results showed that the crystallization rate of ultrasonic-assisted products was the fastest, and the crystallization rate of traditional electric heating methods was significantly lower than that of microwave and ultrasonic methods. Through the step analysis of the synthesis process, they concluded that microwave- and ultrasonic-assisted synthesis can accelerate both nucleation and crystal growth, while the acceleration of crystal growth is more significant [82]. This suggests that ultrasonic and microwave-assisted synthesis may be promising methods to attain MOF more efficiently under mild conditions. Akbarzadeh *et al*. successfully synthesized antibacterial Zn-MOF using Zn (NO_3_)_2_·6H_2_O and 2, 6-pyridine dicarboxilic acid as substrates by the ultrasound-assisted reverse micelle method. In their study, the mixed reaction solution was placed under the condition of ultrasonic irradiation with power of 175 W, temperature of 40°C, and ultrasonic duration of 21 min, and the Zn-MOF crystals were obtained within 30 min. By examining the physical and chemical properties of the products, they proved that the obtained Zn-MOF has the properties of small particle size, large surface area, and mesoporous (size: 41 nm, surface area: 800 m^2^/g, average pore size: 3.4 nm). Meanwhile, they also evaluated the antimicrobial effects of Zn-MOF on six kinds of bacterial biofilms (*Escherichia coli, Staphylococcus aureus, Klebsiella pneumoniae, Salmonella enterica, Bacillus subtilis*, and *Acinetobacter baumannii*), thus confirming the great antibacterial performance of the Zn-MOF synthesized through the ultrasonic-assisted route [28].

From the above examples, we can see that ultrasonic and microwave-assisted synthesis can help us more efficiently synthesize some MOFs with great application performance. However, compared with the widely used solvothermal method, the application of ultrasonic and microwave-assisted synthesis is still relatively limited, and its limitations in various MOF syntheses need to be further confirmed.

#### Electrochemical synthesis

The electrochemical synthesis of MOFs was first patented by the company BASF in 2005 [83]. This synthesis method proposes to directly dip the metal plate as anode into the electrolyte solution containing organic linkers and then trigger the dissolution of the metal anode as a source of the metal ions needed for the continuous introduction of the reaction by applying a constant electric current. Meanwhile, the negatively charged linkers are driven toward the anode under the influence of an electric field and then coordinated with metal ions to form MOF crystals. In this synthesis method, to minimize the redeposition of dissolved metal ions on the cathode, it is usually necessary to introduce protic solvents or relatively strong reducing compounds into the solution. This electrochemical synthesis method cleverly eliminates the anions introduced along with metal ions in the traditional MOF synthesis process using metal salts as ingredients, such as Cl^−^, ClO_4_^−^, and NO_3_^−^ [84]. It has also been proven to be able to continuously and efficiently synthesize MOFs under milder conditions than solvothermal methods, which makes it attract wide attention. Up to now, with the effects of reaction parameters such as solute proportion, solvent, solution conductivity, voltage, and current density on MOF synthesis and the specific reaction mechanism of electrochemical synthesis being more clearly described, several kinds of MOFs have been proven to be able to be obtained through this electrochemical synthesis route of anodic dissolution, such as ZIF-4, ZIF-7, ZIF-8, ZIF-64, HKUST-1, MIL-100(Al), MIL-53(Al) and NH_2_-MIL-53 (Al), and MOF-5 [85–87].

However, after the introduction of electrochemical synthesis technology into the field of MOF synthesis, some other electrochemical synthesis processes of MOFs that are different from the anodic dissolution method mentioned above have also gradually emerged. One of the most representative is the cathode deposition method first proposed by Li *et al*. This method does not involve the introduction of a continuously corroded metal anode as a donor of metal ions. Instead, the choice of anodes is not limited. In the cathode deposition method, the metal salt, organic linker, and so-called probase (NO^3−^ in Li’s case) are dissolved together in the electrolyte solution and react on the cathode surface. The probase in contact with the cathode surface will be reduced and generate OH^−^, and the accumulation of OH^−^ on the cathode surface causes the deprotonation of the linkers. Meanwhile, under the action of electric field, the metal cations are driven toward the cathode and then coordinated with the deprotonated linkers to form MOF crystals. In Li *et al*.’s study, this cathode deposition method has been successfully used for the synthesis of MOF-5 [88].

In addition to the above two electrochemical synthesis methods, the electrochemical synthesis of MOF also includes indirect bipolar synthesis and electrophoretic deposition, which have not been widely applied [89]. At present, the research on the electrochemical synthesis of MOF is still mainly focused on the improvement of the anode dissolution method and cathode deposition method, as well as the application of them in the development of new MOF crystals.

#### Mechanochemical synthesis

Mechanochemical synthesis refers to the use of mechanical forces to induce chemical reactions to achieve the synthesis and transformation of substances. At present, the mechanochemical synthesis of MOF mainly refers to the synthesis of MOF by grinding to promote solid-phase chemical reaction under the condition of no solvent or least solvent. Because the use of organic solvents is largely avoided, and metal oxides and metal hydroxides with low solubility are allowed to replace metal salts as metal precursors for the synthesis of MOF, the mechanical synthesis of MOF produces few harmful by-products, making it the most environmentally friendly process for the production of MOFs. At the same time, the mechanochemical synthesis of MOF has been proven to be able to obtain MOFs with quantitative yield at room temperature relatively quickly. Therefore, as an environmentally friendly, efficient, and sustainable MOF synthesis process, the mechanochemical method possesses great potential in the field of MOF synthesis.

The simplest mechanochemical method used to synthesize MOF is solvent-free grinding (SFG), that is, mechanochemical synthesis under completely solvent-free conditions. For example, in the work of Pichon *et al*., the microporous MOF [Cu(INA)_2_] (INA = isonicotinate) has been successfully developed after grinding the mixture of Cu(O_2_CCH_3_)_2_·H_2_O and isonicotinic acid in solvent-free conditions for 10 min using a ball mill [90]. However, for the synthesis of other MOFs, especially when metal oxides are used as substrates, the mechanochemical synthesis using SFG is often slow and difficult. By introducing a small amount of liquid solvent or liquid containing a little salt additive to catalyze the reaction, the formation of MOF can be accelerated, making the slow and hard mechanochemical synthesis of MOF easier to achieve or more efficient. These two methods of catalyzing mechanochemical synthesis by introducing a solvent or saline solvent are also known as liquid-assisted grinding (LAG) and ion and liquid–assisted grinding (ILAG). For example, the mechanochemical synthesis of MOF using zinc oxide without any catalyst is often difficult to achieve. However, Beldon *et al*. used ammonium salts and a small amount of solvent to promote the inefficient mechanochemical reaction between imidazole and ZnO and achieved topologically selective synthesis of ZIF with quantitative yield within 30–60 min [91]. In fact, for most MOF families, it has been proven that synthesis can be achieved through the above-mentioned three mechanochemical synthesis routes of SFG, LAG, and ILAG. However, the current mechanochemical synthesis of MOF is mostly limited to small volume production, and the application of mechanochemical method in the commercial production of MOFs still needs further investigation.

At present, the synthesis methods of MOFs show diversity in terms of structure regulation and performance optimization, but each has its own advantages and disadvantages. Solvothermal synthesis, as the earliest developed technology, has the advantages of being mature and reliable. By regulating temperature, time, and solvent systems, MOFs of different morphologies and particle sizes can be synthesized. However, its high energy consumption and limited yield limit the potential for clinical transformation. Microwave-assisted synthesis achieves rapid and uniform crystal growth through electromagnetic wave heating, which improves efficiency and safety but has high requirements for the applicability of equipment and reaction systems. Ultrasonic-assisted synthesis has the advantages of low-cost effectiveness, operational safety, and environmental friendliness. At the same time, the generated MOF has a small particle size and high specific surface area, which is suitable for specific applications. Electrochemical synthesis avoids the introduction of anions in the traditional metal salt method and can be continuously synthesized under mild conditions, but it is sensitive to equipment and reaction parameters, and large-scale synthesis technology still needs to be optimized. Mechanochemical synthesis realizes reactions under solvent-free or low-solvent conditions. It is one of the technologies with the greatest environmental potential, and its rapid synthesis characteristics are suitable for small-scale laboratory production. Overall, these synthesis methods have their own advantages in efficiency, environmental protection, and large-scale production capacity, but current research is limited to laboratory synthesis, and large-scale production research has not yet been carried out.

Since the relevant technology is still in the initial development stage, the reproducibility, cost-effectiveness, and environmental friendliness of MOF synthesis methods need to be further focused in future research [92]. In particular, in the field of large-scale manufacturing, the exploration of green synthesis methods is particularly important to develop more cost-effective strategies, reduce dependence on hazardous materials such as toxic organic solvents, and optimize synthesis conditions to avoid harsh manufacturing environments. In addition, large-scale synthesis technology must strictly meet the biocompatibility requirements of biomedical applications. The use of organic solvents and harmful byproducts must be minimized during the synthesis process. Replacing traditional organic solvents with water-based solvents is a direction worthy of attention [93], which can not only significantly improve the biocompatibility of MOFs but also effectively reduce environmental pollution. Through these improvements, not only can the sustainability and safety of MOF materials be improved but also their promotion and clinical transformation in the field of biomedicine will have a positive impact.

### Antimicrobial properties of metal–organic frameworks

Coordination metal ions such as Fe^2/3+^, Cu^2+^, Zn^2+^, Co^2+^, and Ag^+^ in the structure of MOFs can significantly increase the innate cytotoxicity of these materials to bacteria [94]. As mentioned earlier, MOFs have abundant antimicrobial mechanisms. Pure MOFs have made many exciting advances as antimicrobials, as shown in Table 1. In this section, we will focus on silver-based, copper-based, cobalt-based, copper-based, titanium-based, nickel-based, and aluminum-based MOFs as metal-component releasing antimicrobial materials for long-lasting antimicrobial applications.

**Table 1:** Antibacterial effect of pure MOFs antibacterial agent and its mechanism

Metal ion	MOF	Microbial strain	Antimicrobial activity		Antibacterial mechanism	Ref
			ZOI (mm)	MIC (μg ml^−**1**^/ppm)		
Zn^2+^	Zn(formato)_2_(4,4′-bipy)	*E. coli*	–	6	Intracellular ROS production	[95]
		*S. aureus*	–	6		
	Zn(BDC).(H_2_O)	*E. coli*.	16	–	Zn^2+^ release	[96]
		*S. aureus*	14	–		
	BioMIL-5	*S. aureus*	–	1700	Release of AzA and Zn^2+^	[97]
		*S. epidermidis*	–	4300		
Ag^+^	[Ag_2_(bpy)_2_(cbda)]	*E. coli*	12.58	10–15	Stable and sustained Ag^+^ release	[98]
	[Ag4(μ-pydc)2(μ-pm)2]	Methicillin-resistant *S. aureus* (MRSA)	–	256	Ag^+^ release	[99]
		*S. aureus*	–	64		
		*Enterococcus faecalis*	–	256		
		*B. cereus*	–	256		
		*Listeria monocytogenes*	–	256		
		*E. coli*	–	128		
		*P. aeruginosa*	–	128		
		*S. typhi*	–	128		
		*C. albicans*	–	4		
Cu^2+^	Cu/H_3_BTC MOF	*S. aureus*	22	469	Cu^2+^ release	[100]
		*E. coli.*	16	938		
	[Cu(Nic)_2_(H_2_O)_2_]	*E. coli*	2.5	7	The chelation property of metal–organic complex	[101]
		*S. aureus*	3	0.08		
		*P. aeruginosa*	2	2		
Co^2+^	[Co(HL^1^)_2_(4,4′-bipy)]·5H_2_O	*S. aureus*	–	6.25	The chelation property of Co-MOF	[102]
		*C. albicans*	–	12.5		
		*B. subtilis*	–	6.25		
		*E. coli*	–	25		
		*P. aeruginosa*	–	12.5		
Al^3+^	MIL-53(Al)	*E. coli*		128	Particle size, specific surface area, and surface charge of the MOFs	[77]
		*B. cereus*	–	64		
		*S. aureus*	–	32		
		*E. hirae*	–	64		
		*P. aeruginosa*	–	128		
		*Legionella pneumophila* subsp.	–	64		
		*C. albicans*	–	256		
Bi^+^	[Bi(MMTA)3]	*S. aureus*	15	–	The chelation of the ligand with bismuth ion	[103]
		*B. cereus*	19	–		
		*Clostridium butyricum*	19	–		
		*E. coli*	9	–		
		*Enterobacter aerogenes*	7	–		

#### Zn-MOF

As a necessary trace element, zinc itself is involved in many physiological processes in the body. As the constituent and active center of many key metalloenzymes, it plays a vital role in many normal biochemical and metabolic reactions. Evidence has been accumulating about zinc’s role in growing development and the immune response [104]. However, a relatively high concentration of Zn^2+^ has also been proven to show clear antibacterial effects [105]. This antibacterial effect of Zn^2+^ originated from its destruction of microbial cell membrane, induction of ROS production increase, and interference on proteins and DNA [44]. Due to the advantages of biocompatibility and antimicrobial potential, the research on the antibacterial activity of Zn-MOFs is also in full swing.

Currently, many researchers utilize Zn-MOF for antibacterial applications. Akbarzadeh *et al*. developed novel Zn-MOFs nanostructures using the ultrasound-assisted reverse micelle (UARM) method using Zn (NO_3_)_2_.6H_2_O and 2,6-pyridine dicarboxylic acid as substrates. The Zn-MOF nanostructure has good physical and chemical properties and has high antibacterial properties, which can inhibit the growth of six common bacteria including *S. aureus* and *E. coli* [28]. Cao *et al*. obtained three different Zn-based MOFs through the solvothermal method, MOF-5, and ZIF-8. The antibacterial effect of these MOFs was evaluated, and it was observed that both MOF-5 and ZIF-8 had limited antibacterial abilities under the experimental conditions. By analyzing the antibacterial activity and ion release curve of the materials, it was discovered that MOFs with rapid ion release rates exhibited strong bactericidal properties. However, excessively fast ion release could lead to a shorter effective antibacterial duration. Therefore, maintaining an appropriate ion release rate is crucial for the bactericidal performance of MOFs [106]. In addition, other studies also suggest that the antibacterial activity of Zn-based MOFs may not depend solely on ion release. The antibacterial capacity of several different forms of [Zn(dcbp)] (H2dcbp = 2,2'-bipyridine-4,4'-dicarboxylic acid) has been mentioned in the study of Wang *et al*. They examined four different forms of [Zn(dcbp)] (nanoribbons, nanorod flowers, fan-shaped structures, rhombus-shaped structures) and discovered that the fan-shaped particles exhibited superior ability to eliminate Gram-negative bacteria compared to the other forms. This demonstrates that the morphology of the MOF indeed influences its antibacterial activity [107].

It can be seen that the antibacterial properties of Zn-based MOFs have been confirmed in a number of studies. Zinc ions can exert antibacterial effects and promote tissue repair within an appropriate range, but their excessive release may trigger oxidative stress reactions, and excessive accumulation in tissues may cause toxic effects on cells and organs. Although Zn-MOFs (such as the ZIF series) have shown good potential in the biomedical field, their stability and long-term effects in the complex environment of the body still lack systematic verification. Existing biocompatibility studies mainly focus on *in vitro* experiments, exploring their effects on cell viability and short-term toxicity [92]. However, after intravenous or local injection, the *in vivo* distribution, metabolic clearance, and interaction with the immune system of Zn-MOFs still need further study. At present, some studies have shown that Zn-MOFs have good biocompatibility *in vitro*, but their immune risks in chronic use have not been completely ruled out. For example, local or systemic immune responses may occur during long-term use, which puts higher requirements on its safety assessment. Therefore, future studies need to more comprehensively evaluate the *in vivo* biological behavior of Zn-MOFs, including the dynamic release of degradation products, the adaptive response of the immune system, and their long-term toxicity in chronic treatment, to lay a scientific foundation for their clinical application.

#### Ag-MOF

Silver (Ag) is commonly used due to its potent antibacterial properties and low toxicity to eukaryotic cells. The antimicrobial effects of silver are well documented, and it has been utilized for thousands of years in the field of antimicrobial activity. Ag-based antibacterial agents are widely employed for their effectiveness, although they can be costly. Consequently, there is a growing focus on the application of silver-based antibacterial agents in the field of nanomaterials. Silver-based nanoparticles, in particular, have garnered significant attention due to their exceptional antibacterial properties.

Additionally, there is a growing body of research on the antibacterial properties of Ag-based MOFs. Jaros *et al*. first constructed a bioactive MOF, [Ag_4_(μ-PTA)_2_(μ_3_-PTA)_2_(μ_4_-pma)(H_2_O)_2_]n·6nH_2_O (bioMOF 1), constructed from Ag metal nodes and three different bridging ligands. The antibacterial and antifungal properties of this bioactive MOF were further tested. The results showed that bioMOF 1 exhibited superior antibacterial ability than AgNO_3_. Differences in antibacterial ability against different microorganisms were also found. This may be related to the thick cell wall. Related to the hindrance to Ag^+^ penetration. Differences in antibacterial ability against different microorganisms were also found. This may be related to the thick cell wall. Related to the hindrance to Ag^+^ penetration [108]. Liu *et al*. found that Ag-MOFs have stronger antibacterial effects than the most commonly used silver-based antibacterial agents and chemical bactericides previously reported. They further tested their Ag^+^ release characteristics and confirmed that these two products can exert their excellent long-term antibacterial properties through stable and sustained Ag^+^ release [98]. Xia *et al*. compared silver-based MOFs with commercial Ag-NPs (Zone of Inhibition (ZOI) = 11 mm) and found that the Ag^+^ release rate of silver-based MOFs was moderate, and the Ag^+^ release concentration was higher than that of Ag-NPs, indicating stronger antibacterial properties [109]. Lu *et al*.’s study also found that Ag-MOFs have a stronger Ag-releasing capacity than Ag-NPs, and Ag-MOFs exhibit stronger antibacterial effects [110].In the study of Cao *et al*., it was found that p-MOF ([AgL]n·nH2O (L = 4-cyanobenzoate)) has stronger antibacterial ability than two Zn-based MOFs (MOF-5, ZIF-8). This is attributed to two reasons: (i) silver ions are more bactericidal than zinc ions at the same concentration, and (ii) p-MOF’s sustained Ag^+^ release ability leads to its excellent antibacterial activity [106].

The initial antibacterial stage of Ag-MOF is to degrade the MOF structure by releasing metal ions and organic connectors. Silver ions can attach to the bacterial membrane through electrostatic interactions and destroy its integrity [37]. Secondly, Ag^+^, as a sulfophilic element, can increase the intracellular ROS concentration by oxidizing GSH; most importantly, Ag^+^ can specifically bind to sulfhydryl groups and affect protein function [40]. In addition to Ag^+^, there are some areas that need attention. Organic ligands not only affect the release of Ag^+^; carbonyl, carboxyl, amino, and sulfhydryl functional groups can also destroy bacterial cell membranes and cause bacterial cell inactivation [111]. Therefore, the antibacterial activity of Ag-MOF can be improved by adjusting the release of Ag^+^ ions and the type of organic ligands.

Indeed, when compared to other MOFs, the research on Ag-MOFs is more comprehensive in terms of examining the different types of microbial strains and gaining an understanding of their biosafety. This has led to a better understanding of the various types of MOFs. Overall, Ag-MOFs show great potential as antimicrobial agents for practical therapeutic applications. It is believed that once the knowledge regarding the pharmacological and toxicological effects of Ag-MOFs in antibacterial treatment is further enhanced, their antibacterial properties can be fully utilized.

#### Cu-MOF

Just like silver, the antimicrobial application of copper is also very long and extensive. From the past copper products to the present copper ionic antimicrobial agents and copper nanoparticles, the application of copper in the field of antimicrobial agents has been progressing. The antimicrobial effect of these Cu-based antimicrobials is also largely derived from Cu^2+^, which plays a broad-spectrum antibacterial effect in a similar mechanism to Ag^+^. However, unlike Ag^+^, the antimicrobial effect of Cu^2+^ is more likely to be attributed to the direct damage to the cell membrane and proteins rather than the induction of intracellular oxidative stress. Besides, like Zn, Cu is an endogenous trace element with relatively low toxicity, thus possessing the advantage of biological safety. In any case, the application value of copper in the field of antibacterial is very rich, and Cu-based MOF, as an extension of copper in the field of antibacterial application, has also attracted wide attention.

The antimicrobial ability of Cu-based MOFs has been demonstrated in many studies. Shams *et al*. conducted a synthesis of Cu/H_3_BTC MOF and examined its ability to inhibit bacterial growth. The findings indicated that the effectiveness of the product against bacteria was influenced by its concentration. Furthermore, additional experiments utilizing a scanning electron microscope revealed damage to the bacterial cell membrane. Agarose gel electrophoresis results indicated that the DNA of the treated strain was both damaged and degraded. Researchers hypothesize that these observations are a result of Cu^2+^ ions being released from the MOF [100]. Chiericatti *et al*. synthesized HKUST-1 and tested its antifungal activity against *Saccharomyces cerevisiae* and *G. candidum*. This particular substance demonstrated remarkable antifungal properties against both *S. cerevisiae* and *Magnolia alba*, showcasing a bactericidal rate of 99.99%. Additional investigations into the physical and chemical properties of Cu-MOF revealed that it degraded in the culture medium, indicating that the MOF can release Cu ions by breaking down its own structure, thus exerting antibacterial effects [112].

Three Cu-MOFs based on itaconic acid and nicotinamide precursors were constructed in the work of Tella *et al*. The researchers measured the antibacterial activity of several MOFs and found that some Cu-MOFs exhibited stronger antibacterial activity compared to the ligands. Interestingly, the Cu-MOFs did not release any components that could explain their antibacterial effect, as observed in ultraviolet (UV)-visible light studies. Instead, Terra *et al*. proposed that the enhanced antibacterial effect of the Cu-MOFs could be attributed to Tweedy’s chelation theory. According to this theory, the chelating properties of metal–organic complexes increase the lipophilicity of the complex, allowing it to easily penetrate the lipid layer of microbial cell membranes and exert antibacterial effects beyond the potency of the ligands [101]. Yoshizawa *et al*. used four copper salts as precursors to synthesize rod-shaped Cu-based MOF and tested its blood compatibility. The results showed that it did not cause obvious hemolysis or coagulation, proving that this Cu-MOF can potential in biomedical applications [113].

From the above examples, we can see that the various antibacterial mechanisms and antibacterial capacity of Cu-MOF have been elaborated and verified in a number of studies. However, because the application of Cu-MOF is still in its infancy, information about its biological effects in practical applications is still scarce. Further research is needed to clarify important information for specific applications, such as biocompatibility, concrete toxicity, and side effects, the relationship between toxicity and dose, and antibacterial efficacy against various actual infections *in vivo*.

#### Co-MOF

Cobalt has been applied in many fields of biomedicine because of its special biological properties such as antimicrobial, anticancer, anti-oxidation, and angiogenesis promotion [114]. As a component of vitamin B12, cobalt itself plays an important role in preventing anemia and maintaining health. In fact, just like other metal elements, Co can cause the damage or death of microbial cells through a variety of mechanisms to exhibit an antimicrobial effect, and the antimicrobial activity of cobalt and cobalt compounds has also been confirmed in some studies [115, 116]. No exception, cobalt has also appeared in the antimicrobial field of MOFs.

Some studies on the antibacterial performance of Co-based MOF show us its application potential in the antibacterial field. Zhuang *et al*. synthesized a Co-TDM MOF [Co_4_(H_2_O)_2_(TDM)(H_2_O)_8_] (TDM = tetrakis [(3, 5-dicarboxyxy-oxamethyl] methane). They conducted experiments to assess the antibacterial effects of Co-TDM on *E. coli*. The results showed that the bacterial colony was deactivated in <1 h, indicating that Co-TDM possesses powerful antibacterial properties. By employing transmission electron microscopy analysis, they observed that the cell aggregates disappeared and the cell membrane was severely damaged after treating the bacteria with Co-TDM. Based on these findings, it is hypothesized that the main mechanism by which Co-TDM kills bacteria is through damaging the cell membrane [117]. In addition, while the primary mechanism of action for antibacterial activity in Co-MOFs is membrane damage, the actual antibacterial effect is the result of multiple synergistic mechanisms. To understand the antifungal mechanisms of various Co-MOFs, Kim conducted analysis on ROS and reactive nitrogen species (RNS), degradation testing, and antifungal activity testing of ligands and Co^2+^. The findings indicate that the ligand itself does not possess antifungal activity. Although Co-MOFs can generate ROS and RNS and release a certain amount of Co^2+^, these factors alone are insufficient to fully explain the antibacterial ability of Co-MOFs. Thus, Jin *et al*. propose that the antifungal effect of MOF products may arise from the combined impact of a small release of Co^2+^, production of ROS and RNS, and its mechanical damage to bacteria [118].

According to Chinthamreddy *et al*., they discovered that the antibacterial properties of Co-MOFs are a result of the release of Co^2+^ ions. They also observed that the strong intramolecular hydrogen bonds and DABCO coordination between cobalt metal nodes hinder the release of Co^2+^ ions from MOF-2 [119]. On the other hand, Liu *et al*. found that the Co-MOF product exhibited even stronger antibacterial activity compared to the free ligand. They propose that this can be explained by the chelation theory, which enhances the ability of MOFs to penetrate cell lipid membranes and consequently increases their antibacterial effectiveness [102].

Research on Co-MOF is relatively scarce compared with the above-mentioned several metal-based MOFs, but its antibacterial properties and antibacterial mechanism are still well described, which may be due to the support of the study of other MOFs. The antibacterial effects of Co-MOF in more diverse antibacterial situations still need to be further studied. As a metal with a variety of biological properties, the special effects of Co, such as angiogenesis promotion, on its practical antibacterial application effect still need to be further understood.

#### Ti-MOF

The antimicrobial activity of Ti-MOFs is unique compared to other metal-based MOFs. It is believed that this activity is due to their special photocatalytic properties. Substances with photocatalytic activity can generate ROS that have strong oxidation capabilities, which can destroy microbial cells. Some Ti-MOFs, similar to certain metal oxides with semiconducting properties, exhibit excellent photocatalytic activity, which gives them antimicrobial abilities. Several studies have reported on the exceptional antibacterial capabilities of Ti-MOFs.

For instance, Zhou *et al*. synthesized MIL-125(Ti) using titanium isopropoxide and *p*-phthalic acid as substrates. They tested the antibacterial ability of the hybrid membrane MIL-125(Ti)/PVDF against *E. coli* under natural light conditions, and the results showed a 100% antibacterial rate. This confirms the outstanding antibacterial activity of the hybrid membrane produced by MIL-125(Ti) [120]. An NA/NH2-MIL-125(Ti) (NA, 1-naphthylamine) was prepared in the study of Fu *et al*., and they demonstrated its excellent antibacterial properties. They further experimented and found that singlet oxygen is the main active substance involved in the antibacterial activity, which is one of the reactive substances produced by photocatalysis [121]. This further confirms that the antibacterial activity of Ti-MOFs is attributed to their unique photocatalytic activity.

#### Ni-MOF

Nickel is abundant in reserves, widely distributed on the earth, and cheap. The antibacterial activity of Ni-based MOFs has been introduced in several studies. The antibacterial properties of three Ni-based MOFs, [Ni (HL^1^)_2_(2,2′-bipy)]·3H_2_O (MOF-1), [Ni(HL^1^)_2_(4,4′-bipy)] (MOF-2), [Ni_2_(HL^2^)_4_·(4,4′-bipy)·(H_2_O)_2_]·4H_2_O (MOF-3)(H_2_L^1^ = 5-phenyl-1H-pyrazole-3-carboxylic acid; H_2_L^2^ = 3-phenyl-1H-pyrazole-4-carboxylic acid; 2,2′-bipy = 2,2′-bipyridine; 4,4'-bipy = 4,4'-bipyridine) has been mentioned in the study of Liu *et al*. The results indicate that MOFs exhibit higher antibacterial levels compared to free ligands, which is believed to be due to the enhanced chelation effect that increases the ability of MOF compounds to cross the cell membrane [102]. Can *et al*. used Ni as the metal node and the natural amino acid *L*-glutamate to construct the MOF (L-Glu-Ni). After discovering its good antibacterial performance, they further tested the blood compatibility and cell toxicity of the obtained MOFs, and the results showed good blood compatibility and cell compatibility. Overall, Ni-based MOFs have excellent biocompatibility [122].

#### Other metal–organic frameworks

In addition to the several types of MOFs aforementioned, several other metal-based MOFs have also been investigated for their antimicrobial properties. Zafar *et al*. synthesized a nanostructured Mg-based MOF and tested their antibacterial activity. The results showed that the product exhibited stronger antibacterial activity than the ligand, which is thought to be related to the interaction between the metal cation of the product and the negatively charged bacterial cell membrane [123]. In Iram *et al*., four bismuth-based MOFs were synthesized and their antibacterial properties were further evaluated. This product exhibits stronger antimicrobial activity than organic ligands, which is thought to be due to the chelation of the ligands with bismuth ions [103]. The photocatalytic antibacterial ability of vanadium-based MOF was mentioned in the study by Ma *et al*. Visible light irradiation can effectively improve the removal rate of *E. coli* by a vanadium-based MOF. At the same time, experiments also show that the vanadium-based MOF still maintains crystallinity after 1 h of visible light irradiation. This excellent stability indicates that the source of its antibacterial ability is not the release of ingredients, and the product’s quencher treatment test also confirmed its antibacterial performance. Vanadium-based MOF mainly comes from superoxide produced by photocatalytic reaction [124]. Similarly, Zhang *et al*.’s study on aluminum terephthalate–based MOF found that photocatalytic activity is the main source of its antibacterial ability [125].

The antibacterial effect of bimetallic MOFs has also been mentioned in several studies. Chen *et al*. synthesized bimetallic MOF PCN-224 (Zr/Ti) with zirconium and titanium as metal nodes and studied the product’s effect on four common bacteria (*E. coli*, *A. baumannii*, *S. aureus*, *S. epidermidis*) and multidrug-resistant strains [MDR *E. coli*, MDR *A. baumannii*, methicillin-resistant *S. aureus* (MRSA), MRSE]. They found that under light irradiation, the proportion of viable bacteria dropped sharply after only 10 min of incubation with 50 ppm products. After 30 min, the bacteria were almost completely eliminated. The ROS generation induced by efficient photocatalysis is considered to be the main source of the antibacterial ability of this product. In addition, their study further confirmed the excellent biocompatibility of PCN-224 (Zr/Ti), indicating the potential application of PCN-224 (Zr/Ti) in the biomedical field [126]. Li *et al*. studied a bimetallic MOF containing copper and silver. Due to the synergistic antibacterial effect of released Cu^2+^ and Ag^+^, MOFs have excellent antibacterial ability. The synergistic antibacterial effect of bimetals can enable metal ions to achieve strong antibacterial activity at lower concentrations. Their study also demonstrated acceptable cytotoxicity of the MOF at antibacterial concentrations, indicating that Ag/Cu-BTC has sufficient biosafety for practical applications [49].

In this section, we have covered various metal-based MOFs and their antibacterial properties. However, it should be emphasized that although a variety of antimicrobial mechanisms of MOF have been mentioned in the studies we introduced, including component release (metal ions, antibacterial ligands, and modification groups), mechanical damage, surface metal active sites, chelation theory, nanoparticle size effect, and photocatalysis, the antimicrobial mechanism of MOF does not appear to be singular. When MOF is applied to antimicrobial, it often acts through multiple antibacterial mechanisms at the same time, but each has its own emphasis. For example, MOFs constructed on the basis of strong bacterial cytotoxic metals such as Ag, Cu, and Co often rely more on ion release and surface metal active sites to play an antibacterial role. MOFs constructed on the basis of low cytotoxicity or endogenous metals such as Ti, Zr, K, Mg, and Al are more dependent on the release of antibacterial ligands and photocatalysis to show antibacterial activity. Accordingly, the corresponding shortcomings of different MOFs can also be reflected. MOFs based on the release of antibacterial components are often more limited in terms of biosafety in practical applications, while stable and low-toxicity MOFs are often more limited in terms of antibacterial activity and application conditions. Therefore, for a variety of antibacterial MOFs, more studies are needed to clarify their application performance in actual situations, so as to pave the way for their biological application.

### Metal–organic frameworks used as antimicrobial carrier

The inherent microporosity or pores of MOFs enable them to be used as nanocarriers to encapsulate a variety of drugs, thereby producing a synergistic effect. In addition, compared to other porous nanomaterials, such as mesoporous silica [127], the pore size of MOFs can be precisely controlled by the selection of organic ligands and metal ions. This flexibility enables MOFs to load various types of drug molecules and achieve precisely controlled release behavior; secondly, MOFs can exhibit intrinsic antibacterial capabilities while loading drugs, which can not only enhance the antibacterial properties but also further synergize with the loaded drugs. In contrast, porous nanomaterials such as mesoporous silica themselves lack this intrinsic activity and rely more on loaded drugs to achieve therapeutic effects. In fact, there are some attempts have already been reported to apply MOF as a drug carrier in the field of antimicrobial, proving the feasibility of the construction of an MOF-based antimicrobial delivery system, as shown in Table 2. We will introduce the antimicrobial carrier application of MOF in this section.

**Table 2:** Antibacterial effect of pure MOF antibacterial agent and its mechanism

**Composite constituent**		**Antimicrobial substance**	**Role of MOF**	**Advantages of MOF-based** **composite** **antimicrobial agent**	**Ref**
**MOFs**	**Drugs**				
ZIF-8	Ciprofloxacin (CIP)	CIP	Carrier	It has pH-responsive drug release properties that provide precise drug release in acidic environments, resulting in improved antimicrobial efficiency	[128]
ZIF-8	Gentamicin (GEN)	GEN	Carrier	It can combine pH-responsive release with the antimicrobial ability of MOF itself to significantly enhance the antimicrobial effect	[129]
MIL-53(Fe)	Vancomycin (Van)	Van	Carrier	It enhances the antimicrobial activity of VAN, especially in complex infectious settings	[130]
o -NBA@ZIF-8	Rifampicin (RFP)	RFP	Carrier and photoresponsive agent	It can use the light-responsive characteristics to achieve precise and controlled drug release, greatly improving the precision of antimicrobial therapy	[131]
PCN-224	Ag NPs + hyaluronic acid (HA)	Ag^+^ and photogenerate ROS	Carrier and photocatalyst	It combines photocatalysis and drug release to enhance antimicrobial properties and enable precise controlled release	[132]
Cu-TCPP(Fe)	glucose oxidase (GOx)	·OH	Carrier and enzymatic catalyst	It combines pH response with enzyme catalysis to produce hydroxyl radicals for efficient sterilization	[133]

#### Drug loading strategies based on metal–organic frameworks

The importance of combining antimicrobial mechanisms and establishing drug delivery systems to improve the efficacy of existing antimicrobials, especially antibiotics, has been described in the previous section. Therefore, based on the above perspectives, the construction of MOF-based drug delivery systems is a potential way to reverse the bottleneck of current antibiotic treatment. Currently, there are three kinds of drug-loading strategies based on MOFs: encapsulation, assembly, and postsynthesis [134]. Encapsulation is the most commonly used drug-loading strategy, which is based on the porous nature of MOF. In this way, drugs are loaded directly into the pores of the MOF, but it also requires the MOF used to have sufficient pore size to accommodate the encapsulated drug. According to the way of drug encapsulation, it can be further divided into one-step encapsulation and postsynthesis encapsulation. One-step encapsulation refers to the loading of drugs directly in the synthesis process of MOF. However, because the synthesis environment of MOF is often not mild, one-step encapsulation has harsh requirements for the stability of drugs. At the same time, the rate of drug release is slower and more stable. Drug molecules need to overcome physical barriers or chemical binding forces in the framework pores to be released, making them suitable for drug delivery applications that require sustained release. Postsynthetic encapsulation refers to mixing the synthesized MOFs with the drug to realize the loading. Although this method is relatively tedious and time-consuming, it is more commonly used due to its wide application and stable loading rate. The assembly method is a loading approach that directly uses drugs as ligands to participate in the construction of MOFs. Under this loading mode, the release rate of the drug tends to be relatively fast because the drug mostly exists in the form of physical adsorption, which is weakly bound to MOFs and is easily affected by the external environment (such as solution pH or ionic strength). This approach is suitable for applications that require rapid drug release, and the need for long-acting release may be limited. In fact, we have mentioned some antimicrobial MOFs that rely on the release of ligand components in the previous part [97, 135], and their essence is to work as the carrier of active ligands. The advantages of the assembly method lie in the uniform distribution of drugs, high loading capacity, avoiding the introduction of additional ligand, and the product can be used as a carrier to further load drugs, realizing the dual loading of drugs. However, similar to one-step encapsulation, since the drug directly participates in the synthesis of MOF as a ligand, the assembly method has relatively limited requirements on the stability of the drug or the conditions of MOF synthesis [136]. Because the drug molecule is deeply embedded in the MOF, which significantly affects the release rate, its release may take longer, which is suitable for long-term treatment needs. Post-synthesis method uses the metal nodes of MOFs or special functional groups of ligands, such as hydroxyl, carboxyl, and amide, to attach drugs to the MOF through covalent interaction or coordination interaction. This method is often used to functionalize MOF, such as NH_2_-MIL-53(Al) and UiO66-NH_2_ introduced in the previous part [77, 137]. However, due to the fact that the drug molecule is mostly exposed to the surface, the release rate is usually relatively fast, and it has relatively few applications in drug loading compared to the other two loading methods. Based on the above three drug loading methods, a variety of MOFs have been studied for their application value in drug delivery. However, in view of the demand for biocompatibility, some low-toxicity metals (Zn, Fe, Zr) and ligands (imidazole, porphyrin, cyclodextrin) are more selected for the construction of drug-loading MOF carriers [138].

#### Antibiotic delivery

Constructed from Zn metal nodes and 2-methylimidazole ligands, ZIF-8 has attracted considerable attention in the field of antibiotic delivery due to its high porosity, relatively large pore size, high surface area, and excellent biocompatibility. Nabipour *et al*. obtained a ciprofloxacin (CIP)-encapsulated MOF (CIP@ZIF-8) by postsynthetic encapsulation, in which CIP was mixed with the obtained ZIF-8 and stirred at room temperature for 5 days. The drug loading rate of the product was as high as 21 wt%, and the product showed a pH-responsive drug release characteristic, which exhibited a faster drug release under an acidic environment (pH = 5.0) than in physiological conditions (pH = 7.4). Further determination of the antibacterial activity also showed that CIP@ZIF-8 (ZOI: 49 mm for *S. aureus*, 46 mm for *E. coli*) was more effective than that of CIP (ZOI: 25 mm for *S. aureus*, 24 mm for *E. coli*) and ZIF-8 (ZOI: 12 mm for *S. aureus*, 14 mm for *E. coli*) [128]. Similarly, ceftazidime (CAZ)-encapsulated MOF CAZ@ZIF-8 (loading rate: 10.8 wt%) and gentamicin (GEN)-encapsulated MOF GEN@NZIF-8 (loading rate: 19 wt%) have also been prepared [129, 139], which have also been shown to possess pH-responsive drug release properties and antimicrobial abilities stronger than the loaded antibiotics. After loading on MOF, it can promote the internalization of cephalosporin by cells and enhance its intracellular antibacterial effect. They serve as a versatile nanoplatform for *ex vivo* antibiotic delivery and intracellular bacteria eradication.

**Figure 4:**
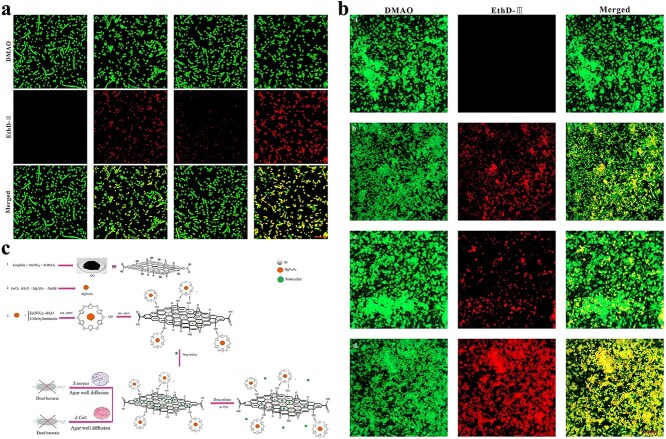
ZIF-8 is used for antibiotic delivery. (**a**) CLSM imaging of death/live staining after *A. baumannii* exposure to various treatments (PBS, imipenem, ZIF-8 and Imi@ZIF-8) for 24 h [140] (reproduced from ref. [140]. Copyright 2023, Frontiers). (**b**) CLSM images of *A. baumannii* biofilms treated with various treatments (PBS, imipenem, ZIF-8, or Imi@ZIF-8) for 24 h [140] (reproduced from ref. [140]. Copyright 2023, Frontiers). (**c**) Simulation of the preparation of the ZIF-8/GO/MgFe_2_O_4_ framework, drug loading, release, and antibacterial tests [143] (reproduced from ref. [143]. Copyright 2021, Springer Nature). *ZIF-8* zeolitic imidazolate frameworks-8, *CLSM* confocal laser scanning microscope

In addition to pure ZIF-8, some studies have attempted to deliver antibiotics using ZIF-8 modified or containing other antimicrobial substances. There are studies on the synthesis of positively charged ZIF-8 for loading negatively charged imipenem antibiotics. The nano-system Imi@ZIF-8 not only has pH-sensitive properties but also has a synergistic antimicrobial effect. As Figure 4 shows, Imi@ZIF-8 had a strong killing effect on *A. baumannii* and could also inhibit the formation of biofilms [140]. Chowdhuri *et al*. applied folic acid (FA), an essential substance for bacterial nucleotide synthesis, to the surface modification of MOF due to its targeting specificity. They further applied the product to vancomycin (VAN) encapsulation, resulting in a ZIF-8@FA@VAN with an antibiotic loading rate of 24 wt%. To evaluate the antibacterial activity of the product, they selected vancomycin-resistant MDR *S. aureus* and MDR *E. coli* for the antibacterial test. The results indicated that the product showed excellent antibacterial activity, while neither MOF nor antibiotics alone showed antibacterial activity against MDR strains. ZIF-8 can promote the uptake of VAN by *E. coli* and *S. aureus* and promote the production of intracellular ROS [141]. Akbari *et al*. fabricated a TiO_2_@Chitosan@ZIF-8 and successfully applied it to the tetracycline (TC) encapsulation. According to their study, the final drug loading rate of TiO_2_@Chitosan@ZIF-8@TC was 87 wt%, and the drug release rate at different pH values also showed significant differences. Further antibacterial tests against *E. coli* and *S. aureus* also indicated that the products showed outstanding antibacterial performance, which was better than tetracycline alone [142]. Similarly, for better TC loading, Saleheh *et al*. incorporated MgFe_2_O_4_ nanoparticles into the synthesized ZIF-8 and then loaded them on the surface of graphene oxide. Follow-up results showed that 90% of the TC was loaded on the synthesized ZIF-8/GO/MgFe_2_O_4_ nanostructure. ZIF-8/GO/MgFe_2_O_4_ not only controlled the release of tetracycline but also improved the antimicrobial properties of tetracycline.

Although it has attracted much of the attention of MOF drug delivery studies, ZIF-8 is not the only option for drug loading of Zn-based MOF. Several other Zn-based MOFs besides ZIF-8 have also been reported for antibiotic loading. Nabipour *et al*. studied the gentamicin delivery application of Zn_2_(bdc)_2_(dabco). Similar to ZIF-8, the product also exhibited significant pH-sensitive drug release properties, with significantly higher antibiotic release levels under acidic conditions (pH 5.0, 65% after 140 h) than in physiological conditions (pH 7.4, 33% after 140 h). The antibacterial activity of the product was also confirmed in the study (*S. aureus*: ZOI = 16 mm, *E. coli*: ZOI = 9 mm) [144]. Linezolid (LNZ) encapsulated MOF LNZ@MOF-74(Zn) prepared by Ramos *et al*. was evaluated for its properties and antibacterial activity against *S. aureus*. The antibiotic loading rate of LNZ@MOF-74(Zn) was reported to be 4.91 wt%, and a pH-responsive controlled release of the antibiotic was also observed. The MIC value of LNZ@MOF-74(Zn) was 75 ppm, containing 3.682 ppm antibiotic, which was significantly lower than that of LNZ (5.5 ppm). It is worth mentioning that LNZ@MOF-74(Cu) constructed with Cu as a metal node was also studied in this study, and the results showed a relatively low drug loading rate (1.75 wt%) and relatively strong antibacterial performance (MIC = 32 ppm, containing 0.112 ppm LNZ), which is thought to be related to the stronger antibacterial effect of Cu than Zn [145]. However, considering that LNZ@MOF-74(Cu) is not as effective as LNZ@MOF-74(Zn) in drug loading and controlled release, as well as potential deficiencies in biocompatibility, its practical application value may not be as outstanding as its antibacterial ability.

**Figure 5:**
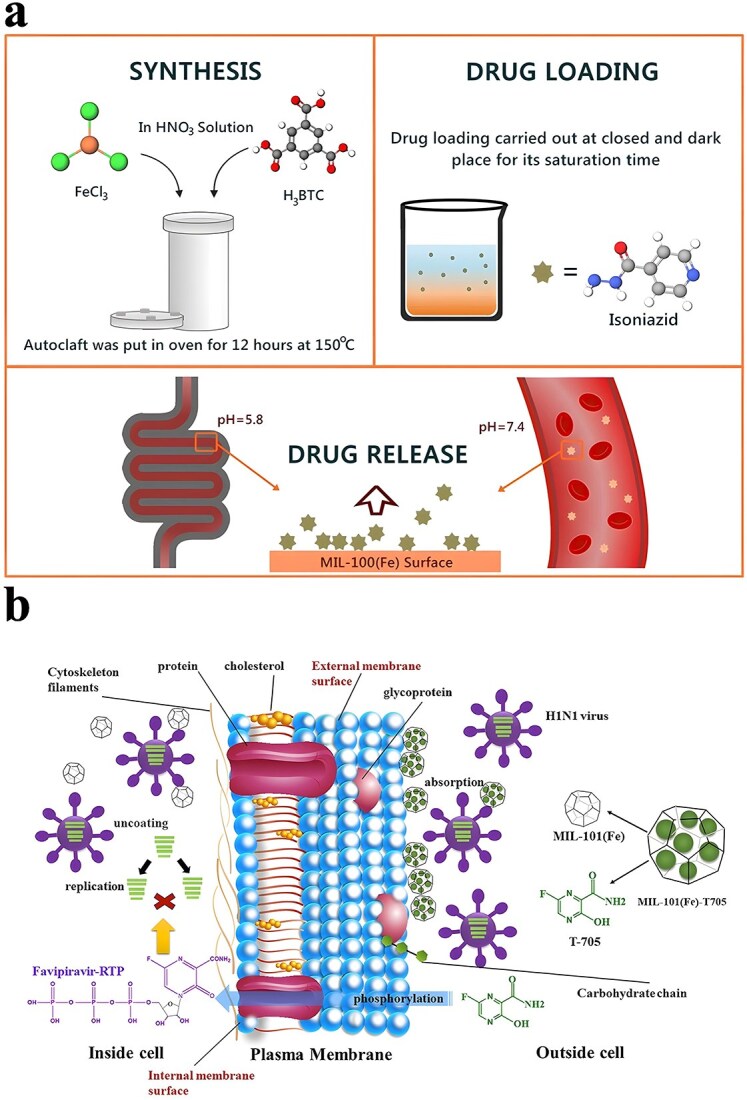
Fe-MOF is used for antibiotic delivery. (**a**) MIL-100 (Fe) as an INH drug delivery platform [148] (reproduced from ref. [148]. Copyright 2021, Springer Nature). (**b**) Nanomaterial MIL-101 (Fe) as a vehicle for the drug favipiravir (T-705) [149] (reproduced from ref. [149]. Copyright 2021, MDPI). MOF, metal–organic framework. MIL-100, materials of institute lavoisier-100. *INH* isoniazid

As another favored metal element also considered to have excellent biosecurity, an Fe-based MOF has also been extensively studied in the field of antibiotic loading application. With iron chloride and terephthalic acid as substrates, Lin *et al*. synthesized MOF-53(Fe) and applied it to the encapsulation of vancomycin (VAN). The obtained MOF-53(Fe)@Van had a high drug loading rate (20 wt%) and showed an accelerated drug release rate with the increase of pH value. Combined with an antibacterial test and cytotoxicity assay, MOF-53(Fe)@Van was shown to exhibit high antibacterial performance (for *S. aureus*: bacteriostatic rate of 99.3%) without cytotoxicity, and the Fe^3+^ released by the degradation of MOF-53 was even confirmed to promote the proliferation and differentiation of MC3T3 cells [130]. Similarly, Haseena *et al*. prepared Fe-MIL-88NH_2_ loaded with VAN and tested its antibacterial activity. Their study showed that the encapsulation efficiency of VAN-Fe-MIL-88NH_2_ was 70.87 ± 2.65%. According to the detection of MIC value against sensitive *S. aureus,* drug-resistant *S. aureus,* and clinical isolation of *S. aureus*, the antibacterial activity of VAN increased after encapsulation (MIC before loaded = 25 ppm vs. after loaded = 10 ppm) [146]. Another study by Ghaffar *et al*. on VAN-loaded Fe-based MOF VAN-MIL-53(Fe) (loading rate of 11.49 ± 2.65 wt%) also obtained similar results, observed the antibacterial ability enhancement of VAN after being loaded (for sensitive *S. aureus*: MIC = 8.5 ± 0.94 ppm; for resistant *S. aureus*: MIC = 18.27 ± 1.09 ppm). They get further using chitosan (CS) to coat the VAN-MIL-53(Fe) and found that the CS-VAN-MIL-53(Fe) antibacterial ability was also further improved (for sensitive *S. aureus*: MIC = 3.81 ± 1.13 ppm; for resistant *S. aureus*: MIC = 8.92 ± 0.69 ppm) [147]. In addition to VAN, there have also been reports of Fe-MOF loading other antibiotics. Isoniazid (INH) is a nitrogen-containing heterocyclic drug known for its antimycobacterial properties in the treatment of tuberculosis; however, its solubility and bioavailability are extremely poor; Simon *et al*. found that MIL-100 (Fe) as an INH drug delivery platform can overcome the above shortcomings [148] (Figure 5a). In another study on Fe-MOF, the nanomaterial MIL-101 (Fe) was compounded with the drug favipiravir (T-705) by solvothermal method to form a new nanocomposite MIL-101 (Fe)-T705. It was found that MIL-101 (Fe) not only promoted the antiviral effect of T-705 but also gave it antimicrobial properties [149] (Figure 5b).

**Figure 6:**
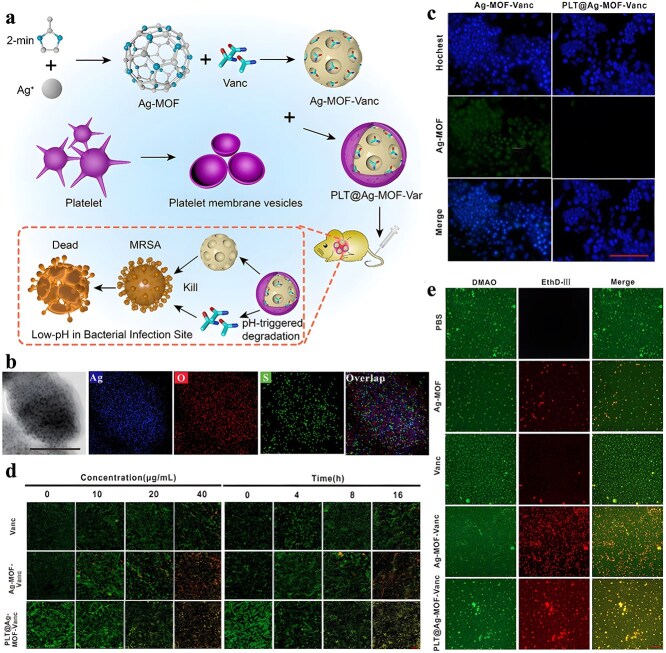
Ag-MOF is used for antibiotic delivery. (**a**) Schematic illustration of PLT@Ag-MOF-Vanc in treatment for MRSA infection [150] (reproduced from ref. [150]. Copyright 2021, Springer Nature). (**b**) TEM image and corresponding element mapping of PLT@Ag-MOF [150] (reproduced from ref. [150]. Copyright 2021, Springer Nature). (**c**) CLSM images of RAW264.7 cells upon culture with Ag-MOF-Vanc or PLT@Ag-MOF-Vanc for 24 h [150] (reproduced from ref. [150]. Copyright 2021, Springer Nature). (**d**) CLSM imaging of MRSA death/biostaining after exposure to Vanc, Ag-MOF-Vann, or PLT@Ag-MOF-Vanc at different concentrations and different incubation times [150] (reproduced from ref. [150]. Copyright 2021, Springer Nature). (**e**) CLSM images of MRSA biofilm [150] (reproduced from ref. [150]. Copyright 2021, Springer Nature). *MRSA* methicillin-resistant *S. aureus*, *Vanc* vancomycin, *PLT* platelet, *CLSM* confocal laser scanning microscope

In addition to the relatively common MOF carrier options described above, there are several antibiotic delivery studies about other MOFs have been reported. Huang *et al*. loaded vancomycin (Vanc) with Ag-MOF and then encapsulated Ag-MOF-Vanc with nano-sized platelet vesicles to develop platelet membrane-camouflaged nanoparticles PLT@Ag-MOF-Vanc for the treatment of MRSA infection. PLT@Ag-MOF-Vanc can effectively improve the antimicrobial activity of vancomycin and can avoid macrophage phagocytosis [150] (Figure 6). UiO-66 constructed from low-toxic metal Zr and the ligand 1,4-benzene dicarboxylic acid (BDC) was prepared for the delivery of ciprofloxacin (CIP) in Nasrabadi *et al*. The obtained CIP-UiO-66 had excellent drug loading performance (loading rate of 84 wt%), and the drug release of the product showed pH-responsive property. The ZOI of the product against *S. aureus* and *E. coli* was also tested, and the results showed that CIP-UiO-66 exhibited stronger antibacterial activity than CIP (*S. aureus*: ZOI = 22 mm vs. 0 mm of CIP; *E. coli*: ZOI = 24 mm vs. 14 mm of CIP) [151]. Another study by Chen *et al*. also on Zr-based MOF carrying antibiotics took a different strategy of drug loading. In their study, vancomycin was attached to the surface of porphyrin MOF PCN-224 by a postsynthetic method, resulting in vancomycin surface functionalized VAN-PCN-224. They applied the product to antibacterial tests against *S. aureus* and found that the product showed excellent antibacterial abilities under light conditions (after 20-min LED light irradiation: bacterial viabilities: 14.2% of 20 ppm, 0.9% of 60 ppm), while under dark conditions of products thought to derive mainly from vancomycin itself (bacterial viabilities: 56.9% of 20 ppm, 16.6% of 60 ppm) and antibacterial ability of PCN-224 itself under light irradiation (bacterial viabilities: 75.1% of 20 ppm, 19.4% of 60 ppm) were relatively weak [152].

#### Inorganic antimicrobial delivery

The most common MOF-loaded inorganic antimicrobial cargo reported in studies is Ag, which may benefit from its outstanding antibacterial ability compared to other metals and its relative maturity in application. Huang *et al*. successfully obtained Ag@MIL-53(Fe) by impregnating the prepared MIL-53(Fe) in an AgNO_3_ solution. Assessment of antibacterial activity showed that MIL-53(Fe) had no significant antibacterial activity on *S. aureus* and *E. coli*, while Ag@MIL-53(Fe) showed antibacterial activity related to the loading rate of Ag. Further cytotoxicity tests also demonstrated excellent biocompatibility of Ag@MIL-53(Fe) at concentrations below 20 ppm [153]. In a similar way, Li *et al*. synthesized Ag@MIL-53(Fe) (Ag 0.0127 wt%). Through the further determination of product properties, Ag@MIL-53(Fe) was confirmed to have antibacterial activity against the six bacteria tested, and the cytotoxicity and hemolysis tests also showed that Ag@MIL-53(Fe) possessed great biocompatibility [154].

However, the Ag cargo used for MOF loading is not limited to silver ion compounds, Ag NPs are another common form of Ag cargo. Ag-CUTCPP MOFs containing Ag NPs were prepared by Guo *et al*. by one-step encapsulation, and their antibacterial abilities against three bacteria were confirmed in further antibacterial tests [155]. By the same method, another Ag NP-loaded MOF Ag NPs@Cu/GMP was synthesized in the study of Soomro *et al*. In the further test of antibacterial performance, Ag NPs@Cu/GMP was found to have stronger antibacterial activity than Cu/GMP and Ag NPs, and it was believed that the source of this product’s great antibacterial activity is the release of Ag NPs and Cu ions and the synergistic effect of the two [156]. Different from the above-mentioned loading methods of silver cargo, in Wu *et al*.’s study, the silver cargo was loaded on the MOF through the postsynthesis method. They designed Zn-MOF with reserved terminal alkene functional groups bound to Ag to achieve uniform dispersed loading of Ag in Zn-MOF, and the results of the antibacterial test demonstrated the excellent antibacterial ability of the obtained Ag@Zn-MOF [157]. This reserved site loading method make Ag loading more controllable, and make it possible to further adjust the release of Ag cargo.

**Figure 7:**
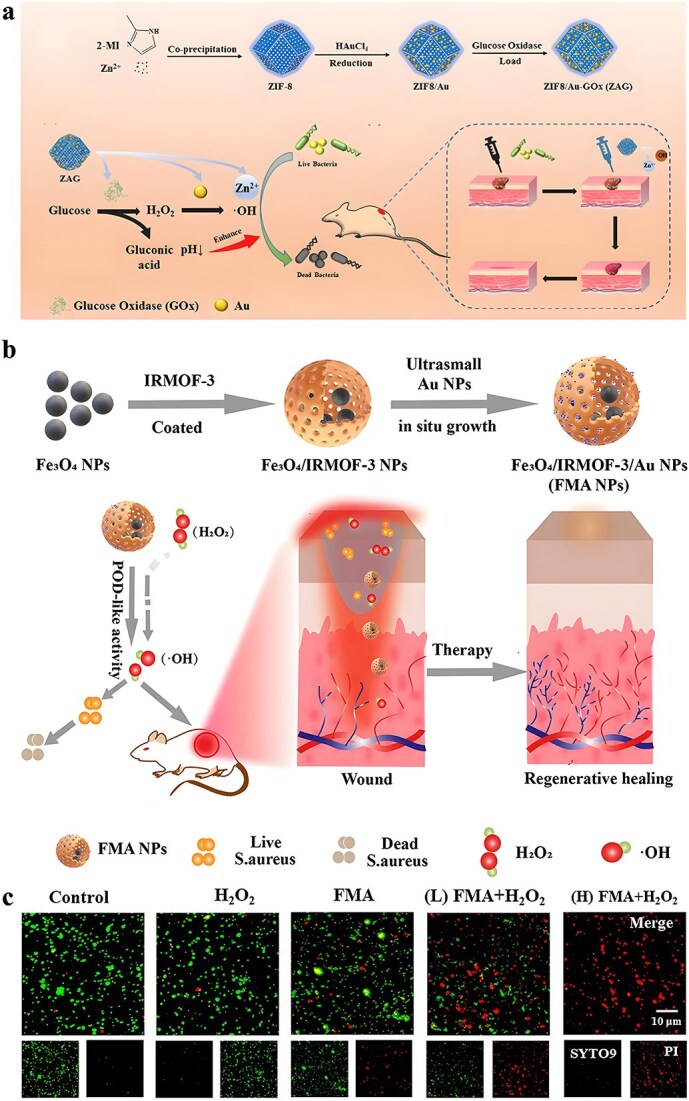
Au cargo used for MOF loading. (**a**) Illustration of the synthesis of ZIF8/Au-GOx NPs and reaction mechanism of the effective acid-enhanced dual-modal strategy for antibacterial treatment [158] (reproduced from ref. [158]. Copyright 2022, Elsevier). (**b**) Schematic diagram of the preparation of IRMOF-3-stabilized FMA hybrid nanozymes and the mechanism of antibacterial wound healing based on high POD-like activities of FMA nanozymes *in vivo* [159] (reproduced from ref. [159]. Copyright 2023, Springer Nature). (**c**) Fluorescence images of live/dead *E. coli* with different treatments [159] (reproduced from ref. [159]. Copyright 2023, Springer Nature). *FMA* Fe3O4@MOF@Au, *POD* peroxidase, *NPs* nanoparticles

In addition to silver, several other inorganic cargos have also been reported for antimicrobial studies of MOF drug loads. In a study, gold nanoparticles (Au NPs) were also loaded on MOFs, and an ROS-based antimicrobial strategy was developed. Wang *et al*. selected ZIF8, which can release Zn^2+^, as a carrier, and integrated glucose oxidase (GOx) and Au NPs to generate ROS through a cascade catalytic reaction. The nanosystem integrates the bactericidal capabilities of ROS and Zn^2+^ to further improve the overall antimicrobial performance [158] (Figure 7a). In another study, MOFs were loaded with both AuNPs and Fe_3_O_4_ NPs. Liu *et al*. constructed heterogeneous bimetallic composite nanozymes Fe_3_O_4_@MOF@Au NPs (FMA NPs) (Figure 7b). In the presence of low doses of H_2_O_2_, the FMA nanocatalyst induces ROS production in bacteria, thereby significantly reducing bacterial growth [159] (Figure 7c).

Nitric oxide (NO) shows positive effects in antimicrobial applications due to its nitrosylation and oxidation effects. However, the short lifespan and reactivity to various biological substances limit the clinical application of NO. Li *et al*. prepared nano-sized Cu-BTC MOFs for the storage and release of NO and used them for antimicrobial activity determination. In fact, NO can be easily stored in Cu-BTC because it can bind to the open metal site of MOF, but this tight chemisorption also makes it difficult to release the stored NO. However, in the study of Li *et al*., NO release was regulated by adjusting sample morphology, hydration degree, and NO charging pressure so that the obtained NO@Cu-BTC NO release characteristics could meet the needs of therapeutic applications. The results of antibacterial tests in the study showed that NO@Cu-BTC exhibited significant antibacterial activity and was stronger than Cu-BTC itself, this indicates that NO@Cu-BTC can play a synergistic antibacterial effect through the release of NO, which represents the feasibility of using MOF as NO carrier for antibacterial therapy [160].

#### Drug delivery systems with environmentally responsive properties

The double-edged sword of metal ions (side effects) may raise concerns: while the release of metal ions is extremely important for antibacterial effects, excessive metal ion release from MOFs may also damage healthy cells [161]. Therefore, MOF-based materials with stable and controllable metal ion release are needed—to preemptively avoid unintended damage to healthy tissues. We have emphasized in the previous section that in addition to the drug-loading capacity of MOF, its own physiochemical properties and designability are also great advantages for drug delivery. By utilizing this advantage, a targeted, controllable, and efficient drug transport system can be designed on the basis of drug loading. The most common design concept is to build an MOF drug delivery system based on the environmental response characteristics of MOF or to give MOF an environmental response ability. For example, pH-responsive drug delivery systems can be built by the utilization of the properties of some MOFs to accelerate decomposition under acidic conditions [162–164], and targeted drug delivery systems can be built based on the addition of magnetic shells or protein modifications to the surface of the MOFs [34, 141, 152, 165]. In fact, based on these common ideas, several studies have developed their own novel and sophisticated antimicrobial MOF delivery systems.

A light-triggered pH-responsive drug delivery system RFP&o-NBA@ZIF-8 was reported in the study of Song *et al*. They introduced 2-nitrobenzaldehyde(*o*-NBA), a pH-jump reagent, into ZIF-8 and used it to load rifampicin (RFP). In theory, *o*-NBA introduced into the system under the irradiation of specific wavelengths of light will undergo the intramolecular excited-state hydrogen transfer to produce nitrobenzoic acid and protons, thereby reducing the pH value of the environment, accelerating the decomposition of ZIF-8 carrier and releasing the RFP contained therein. This process was confirmed by the determination of environmental pH value and Zn^2+^ release efficiency in the study. In addition, it was found that the decomposition of RFP&o-NBA@ZIF-8 and drug release behavior was highly correlated with the irradiation time, and it was feasible to achieve precise antibiotic release by controlling the light exposure duration. Further *in vivo* and *in vitro* antimicrobial performance tests showed that the product exhibited excellent antimicrobial performance under light conditions, which is thought to be possibly due to the synergistic effect of Zn^2+^ and RFP. The great antibacterial ability and controllable drug release indicated the great advantages of this drug delivery system for application and has the potential to become an effective way for infection treatment [131].

The sensitivity of coordination bonds to external pH variations and the pH fluctuations within the tumor microenvironment serve as triggers for controlled drug release [166]. The sensitivity of some MOFs to pH stems from the chemical stability of the materials in which they are constituent. For example, Zn-MOFs are protonated in an acidic environment, resulting in structural changes and drug release. ZIF-8 itself is pH sensitive. Therefore, He *et al*. synthesized a novel AgNPs@ZIF-8 nanocomposite by taking advantage of the beneficial properties of the low-cytotoxicity carrier ZIF-8 and antimicrobial AgNPs. AgNPs@ZIF-8 has excellent antimicrobial activity compared with AgNPs alone. In addition, AgNPs@ZIF-8 has also been demonstrated to show significant efficacy in eradicating pathogenic bacterial biofilms and inducing elevated levels of ROS within bacterial cells [167].

Tan *et al*. successfully encapsulated the natural organic compound Physicon (Phy) and silver nanoparticles in ZIF-8 and constructed Ag-Phy@ZIF-8@HA NPs after further functional modification by hyaluronic acid (HA). This MOF design enables the product to exert efficient and precise antibacterial action because the hyaluronidase secreted by bacterial growth will cause the product to gather around the bacteria, while the acidic environment generated by bacterial metabolism will further accelerate the decomposition of the product to release Ag NPs and Phy and finally play an antibacterial role at the bacterial colonization site. In fact, the great antibacterial activity and excellent biocompatibility of Ag-Phy@ZIF-8@HA NPs have already been confirmed in their study, which means the great potential for practical antimicrobial applications of this design (for *E. coli:* MIC = 250 ppm, for *S. aureus:* MIC = 130 ppm) [168].

Another gallium- and lyticase-based antifungal infection drug delivery system with a similar structure, namely, MLPGa, was constructed by He *et al*. They also first used MSN loaded lyticase as the core of the system, then coated the surface with an outer layer of MOF structure with Ga as metal nodes and polydopamine as linker, and finally obtained an antifungal drug delivery system with the lyticase drug delivery core and Ga-based MOF shell. The MOF shell of MLPGa constructed by He *et al*. will accelerate decomposition to release Ga^3+^ under the acidic environment of fungal infection, and the MSN carrier as the core will release the loaded lyticase, producing synergistic antibacterial effects. The antifungal ability of the MLPGa system in their study showed that the MLPGA system had a strong antifungal ability (For *Candida albicans*: MIC = 40 ppm, survival rate nearly 0%), and the effectiveness of the product was also confirmed by *in vivo* experiments on fungal keratitis models. Further biocompatibility tests showed that MLPGa still had excellent biocompatibility even at eight times the therapeutic dose (320 ppm), suggesting that this drug delivery system may be a feasible clinical choice for fungal corneal infections [169].

**Figure 8:**
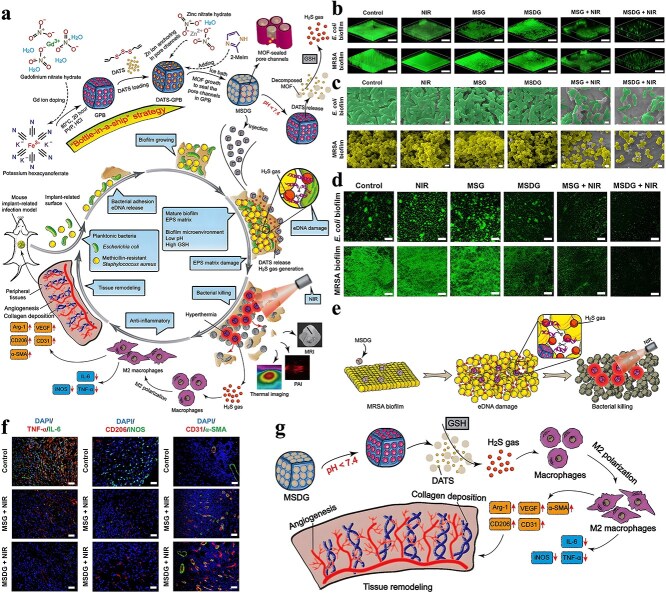
MOF-based bioresponsive nano-antibacterials. (**a**) Schematic illustration of design, synthesis, and biomedical applications of MSDG [170] (reproduced from ref. [170]. Copyright 2022, American Association for the Advancement of Science’s). (**b**) Three-dimensional reconstructions of the fluorescence-labeled *E. coli* and MRSA biofilms stained with Syto9 (reproduced from ref. 170. Copyright 2022, American Association for the Advancement of Science’s). (**c**) High-resolution SEM images of *E. coli* and MRSA biofilms treated with control, near-infrared (NIR), MSG, MSDG, MSG + NIR, and MSDG + NIR [170] (reproduced from ref. 170. Copyright 2022, American Association for the Advancement of Science’s). (**d**) SYTOX staining of eDNA in *E. coli* and MRSA biofilms [170] (reproduced from ref. [170]. Copyright 2022, American Association for the Advancement of Science’s). (**e**) Schematic illustration of the mechanism of biofilm elimination by H2S-sensitized hyperthermia [170] (reproduced from ref. [170]. Copyright 2022, American Association for the Advancement of Science’s). (**f**) Immunofluorescent costaining of TNF-α/IL-6, CD206/iNOS, and CD31/α-SMA [170] (reproduced from ref. [170]. Copyright 2022, American Association for the Advancement of Science’s). (**g**) Schematic illustration of MSDG-mediated improvement of immune microenvironment and tissue remodeling [170] (reproduced from ref. [170]. Copyright 2022, American Association for the Advancement of Science’s). MRSA, methicillin-resistant *S. aureus*. *MSDG* metal–organic framework–sealed Prussian blue–based nanocarrier, *eDNA* extracellular DNA, *NIR* near-infrared, *H*_*2*_*S* hydrogen sulfide

In addition to the above unique design of several antimicrobial MOF drug delivery systems, Su *et al*. proposed a new strategy of “gas-sensitized hyperthermia” (Figure 8a). It kills bacteria through the intelligent design of MOF-sealed Prussian blue–based nanocarriers (MSDGs). Once the biofilm microenvironment (BME) is reached, acidity-activated MOF degradation enables the release of diallyl trisulfide to react with overexpressed GSH (Figure 8e). Under near-infrared irradiation, MSDG-induced H_2_S-sensitized hyperthermia can effectively eliminate biofilms through H_2_S-induced extracellular DNA damage and heat-induced bacterial death. (Figure 8b–d) Subsequently, in the implant-associated infection model, MSDG stimulation promoted macrophage M2 polarization (Figure 8f) secreted abundant regeneration-related cytokines, and accelerated tissue remodeling in the implant-associated infection model [170] (Figure 8g).

In addition to chemical responses, some physically responsive MOF-based materials have also been studied. Some MOFs have excellent sonodynamic effects when stimulated by ultrasound (US). Under US stimulation, MOFs can produce ROS, mainly singlet oxygen (^1^O_2_) and hydroxyl radicals (•OH), which are highly cytotoxic to various drug-resistant bacteria [171, 172]. At the same time, due to its advantages such as noninvasiveness, excellent tissue penetration, and limited ultrasound site irradiation, this shows great potential in the treatment of deep infections, thereby producing antibacterial effects [173, 174]. Here, ZIF-8-derived carbon@TiO_2_ nanoparticles (ZTNs) were developed as inhalable sonosensitizers for bacterial pneumonia. MOF-derived ZTNs have a special structure and have good ROS generation ability under US irradiation. *In vitro* experiments found that ZTNs had excellent inhibition rates against Gram-negative MDR bacteria *K. pneumoniae*, *A. baumannii*, *Pseudomonas aeruginosa,* and *K. aerogenes* under ultrasound irradiation. After US irradiation, ZTNs can effectively eliminate MDR *K. pneumoniae* in BALB/c, server combined immune-deficiency (SCID), and NOD/SCID mouse lung infection models and achieve a 100% survival rate in lethally infected NOD/SCID mice. This strategy provides a useful tool for the treatment of deep-tissue bacterial infections and shows great promise in clinical translation [175].

### Metal–organic framework–based phototherapy

While MOFs have demonstrated advancements in antimicrobial, it is worth noting that prolonged use of metal ions can also lead to bacterial tolerance [176]. As a result, there is a pressing need to explore and create novel sterilization mechanisms. Phototherapy refers to the treatment routes performed under relatively harmless light irradiation conditions. Phototherapy has been extensively utilized in the management of tumors [177], and its research and application in antibacterial has gradually attracted attention. According to the mechanism of phototherapy, it can be mainly divided into photodynamic therapy (PDT) and photothermal therapy (PTT). Although both are under light irradiation, PDT mainly relies on a photosensitizer (PS) to produce ROS that can exert therapeutic effects in the presence of molecular oxygen, while PTT mainly relies on photothermal agents (PTAs) to release heat to exert therapeutic effects [178]. For example, in the previously mentioned content, the photocatalytic effect of some metal oxides and the special antibacterial ability of Ti-based MOF under light irradiation belong to PDT, while the photothermal antibacterial effect of precious metals belongs to PTT. As a therapy that is effective against almost all microorganisms and cannot be resisted, phototherapy has a very important value in the field of antimicrobial therapy [179]. Taking advantage of the light efficiency of some MOFs, easy modification, and drug delivery, there have been a lot of antibacterial phototherapy studies based on MOFs in recent years [180], as shown in Table 3.

**Table 3:** Antibacterial phototherapy studies based on MOFs

**MOF-based phototherapy**	**MOFs**	**Photosensitizer** **(PS)**	**Advantages of MOF-based** **phototherapy**	**Ref**
Pure/Modified MOFs	Prussian blue (PB)@MOF	Porphyrin-based MOF	It combines the properties of MOF and PB to significantly outperform the performance of a single ingredient in its antimicrobial power.	[181]
	TRB-ZnO@G	Carbonized MOF	It has a strong local multibactericidal capacity and can effectively provide treatment against drug-resistant strains.	[182]
	HKUST-1	Cu-MOF after sulfuration	After vulcanization, it exhibits excellent photocatalytic and photothermal properties for antimicrobial treatment.	[183]
	ZnP-CNP-TRGL	Carbonized ZIF-8	It has a super high bactericidal effect on *S. aureus* and *E. coli.*	[184]
	PCN-224-Ag-HA	Porphyrin-based Ag-MOF	It provides precise and long-lasting antimicrobial properties through the controlled release of silver ions.	[132]
MOF loaded PS	RB@ZIF-8	Bengal Red (RB)	It enables targeted drug delivery to microbial cells while reducing the development of drug resistance.	[185]
	Ag@MOF@PDA	Polydopamine (PDA)	It can be combined with silver ion release and polydopamine coating to provide synergistic antimicrobial power.	[186]
	ICG @ Fe-101	Indocyanine green (ICG)	It significantly improves the stability of ICG and enhances the efficacy of photosensitive therapy.	[187]
	ZIF-ICG@ZIF-GOx@MPN	ICG	It breaks the limits of pH and hydrogen peroxide, enabling more effective antimicrobial treatments.	[188]

**Figure 9:**
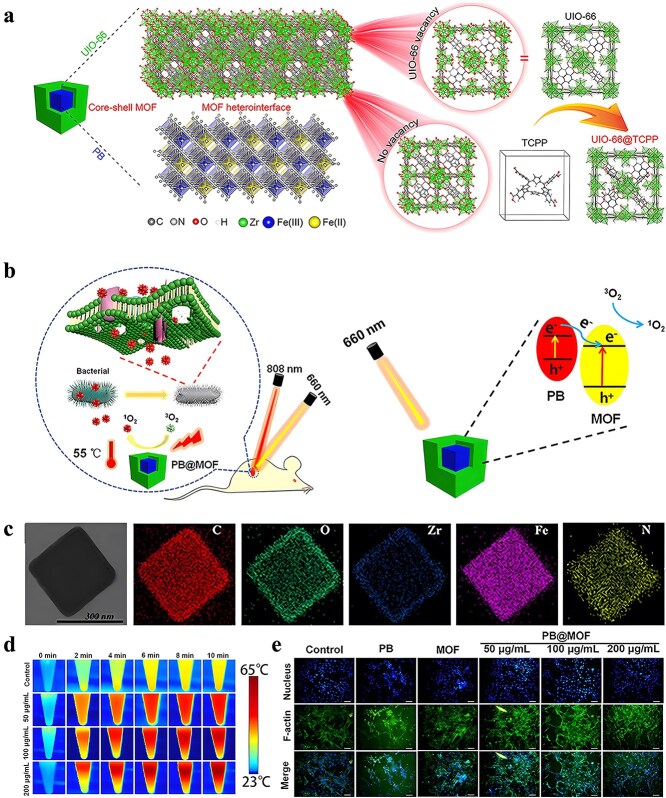
The sterilization effect of PB@MOF particles. (**a**) Schematic illustration of the core–shell structure of PB@MOF [181] (reproduced from ref. [181]. Copyright 2019, ACS publications). (**b**) Schematic illustration of the bacteria-killing processes with the PB@MOF under dual light irradiation [181] (reproduced from ref. [181]. Copyright 2019, ACS Publications). (**c**) EDX elemental mapping images of PB@MOFs [181] (reproduced from ref. [181]. Copyright 2019, ACS Publications). (**d**) Real-time infrared thermal images for different concentrations of PB@MOF solution under 0.5 W cm^−2^ 808 nm NIR for 10 min [181] (reproduced from ref. [181]. Copyright 2019, ACS Publications). (**e**) Fluorescent images of cells after cultivating with different materials [181] (reproduced from ref. [181]. Copyright 2019, ACS publications). *PDT* photodynamic therapy

Some MOFs can generate radicals and heat under light and efficiently kill bacteria. By using PB as the core and wrapping it with a layer of porphyrin-based MOF, a dual MOF system with a core–shell structure (PB@MOF) is formed. Under dual irradiation of 660 and 808 nm, the sterilization effect of PB@MOF particles on *S. aureus* and *E. coli* can reach 99.31% and 98.68%, respectively, significantly higher than pure PB or MOF alone (Figure 9). This is mainly because the electrons generated by the core PB under light can transfer to the outer MOF through porphyrin, inhibiting the recombination of electrons and holes, effectively improving the photocatalytic ability of the composite particles and enhancing their ability to generate radicals under light. Therefore, PB@MOF has the strongest sterilization ability under light [181].

**Figure 10:**
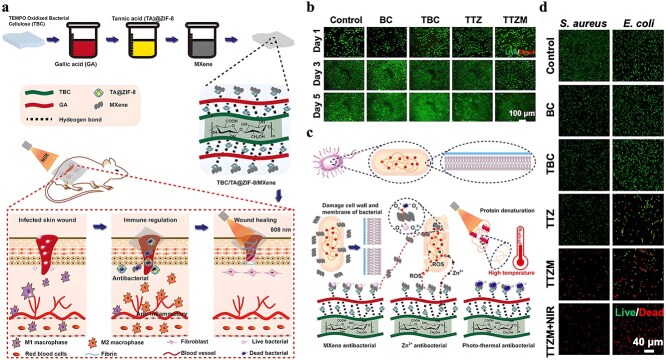
The characteristics of MXene@Zn-MOF-decorated oxidized BC-based hydrogel (TTZM). (**a**) Schematic illustrations of synthesis of TTZM and the mechanism for repairing the infected wounds [189] (reproduced from ref. [189]. Copyright 2023, Elsevier). (**b**) Live-/dead-cell staining images of L929 cells measured from the live/dead images of different hydrogels cocultured with L929 cells for 1, 3, and 5 days [189] (reproduced from ref. [189]. Copyright 2023, Elsevier). (**c**) Potential antibacterial mechanisms of the TTZM hydrogel photothermal antibacterial platform [189]. (reproduced from ref. 189. Copyright 2023, Elsevier). (**d**) Confocal images of *S. aureus* and *E. coli* co-incubation with different hydrogels by using SYT09/PI fluorescent staining [189] (reproduced from ref. [189]. Copyright 2023, Elsevier). *TTZM* MXene@Zn-MOF-decorated oxidized BC-based hydrogel, *TA* tannic acid, *BC* bacterial cellulose, *GA* gallic acid, *SEM* scanning electron microscopy, *TEM* transmission electron microscopy

Some MOFs themselves do not possess photodynamic or photothermal properties, but they can be used as carriers to load photosensitizers, improving the stability and dispersibility of the photosensitizers to enhance their therapeutic effects. Indocyanine green (ICG) is a small-molecule organic photosensitizer that can be adsorbed and loaded onto Fe-based MOF (Fe-101). After being loaded into MOF particles, the stability of ICG is greatly improved, with a storage time extended to over 10 days, and the bactericidal performance is also significantly enhanced. Under irradiation of light at a wavelength of 810 nm, Fe-101 loaded with ICG can achieve a killing ability of 89% against *E. coli* [187]. However, when ICG is loaded onto ZIF-8, synergistic photothermal and metal ion antibacterial effects can be achieved. ICG can produce heat and singlet oxygen (^1^O_2_) under near-infrared laser irradiation, and locally generated heat can kill bacteria and accelerate the conversion of H_2_O_2_ to ·OH; The ^1^O_2_ produced at the same time can also help kill bacteria to achieve PDT. The prepared antibacterial nanoreactor can break the limitation of pH and H_2_O_2_ at the same time, which provides a new idea for the eradication of pathogenic bacteria by combination therapy [188]. Additionally, besides ICG, MXene also shows promise due to its high conversion efficiency of light into heat, biodegradability, and low cytotoxicity. Cheng *et al*. reported a TEMPO-oxidized bacterial cellulose (TBC)-based hydrogel with a tannic acid (TA)-modified metal–organic framework (TA@ZIF-8 [TZ]) with further loading of MXene (TBC-TA@ZIF-8-MXene [TTZM]) (Figure 10a). The hydrogel has good biocompatibility (Figure 10b) and can achieve a highly effective and rapid antimicrobial effect when exposed to 808 nm NIR light [189] (Figure 10c and d).

At present, there are two main problems with phototherapy. One is weak penetration, that is, it has little effect on the lesions deep in the body, and the other is weak selectivity, which makes it difficult to have a targeted effect on the lesions [179, 190]. For the former, PS and PTA with stronger light absorption capacity and higher conversion efficiency are currently being developed to improve the therapeutic depth of phototherapy [178, 191, 192]. For the latter, some MOF-based phototherapy strategies offer good solutions.

### Antimicrobial applications of metal–organic framework

**Figure 11:**
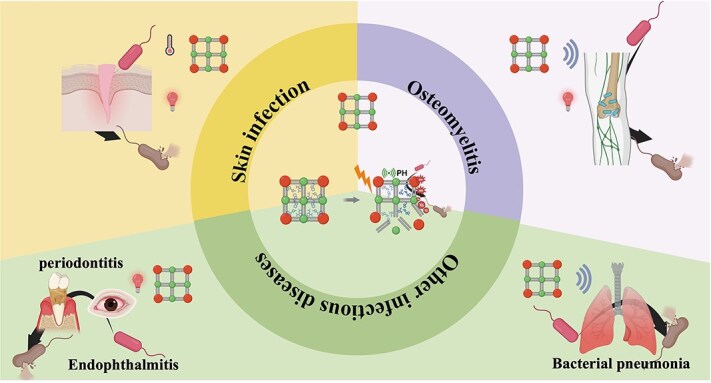
Antimicrobial applications of MOF. *MOF* metal organic framework

In recent years, MOFs have gained significant attention in the fields of drug delivery and catalysis. Their remarkable features, such as a large specific surface area, high porosity, and strong design capabilities, give them numerous advantages. As mentioned before, MOFs possess easily degradable metal sources and organic ligands and good biocompatibility and exhibit effective antibacterial abilities in laboratory experiments. Furthermore, certain types of MOFs exhibit excellent photothermal properties. The development of new multimodal combined antibacterials that can be used in conjunction with traditional photothermal and other physical stimuli has demonstrated a broad-spectrum ability to kill bacteria and has shown immense potential as therapeutic agents for drug-resistant bacterial infections [94]. Currently, there are ongoing studies focusing on the use of MOFs for diseases caused by specific bacterial infections (Figure 11).

#### Skin infections

As the largest organ in the human body, the skin serves several important functions, including protection, sweat excretion, sensing external pressure, and temperature regulation. However, being located on the body’s surface makes the skin vulnerable to injuries [193]. When the skin is damaged, its ability to fight off bacteria is reduced, making it easier for infectious wounds to develop and leading to long-term inflammation. Additionally, as bacteria become more resistant to antibiotics, bacterial infections can worsen skin wounds, preventing them from healing and potentially causing serious complications [194–196] like staphylococcal scalded skin syndrome caused by *S. aureus*. Furthermore, due to the weakened resistance of the body, clinically bacterial skin infections are most common in diabetic patients; skin lesions can aggravate the condition of diabetic patients and even progress to sepsis and death [197]. Therefore, the ability to quickly and effectively antibacterial has become the key to high-quality treatment of infectious wounds. Some MOFs have been used to repair skin infections, as shown in Table 4.

**Table 4:** MOFs and their antibacterial mechanisms are used to repair skin infections

**Composite constituent**		**Role of MOF**	**Animal models**	**Antibacterial mechanism**	**Ref**
**MOF**	**Other** **(antibiotics, PS, etc.)**				
ZIF-8	Mn/Fe	Carrier	*S. aureus*-infected wound Balb/c mouse	Catalyze the production of ROS	[198]
ZIF-8	RFP; *o*-NBA	Carrier	MRSA and ampicillin-resistant *E. coli* infected wound Balb/c mouse	UV, pH trigger precise release of antibiotics and zinc ions	[131]
Bio-MOF	Quaternary ammonium salt chitosan (QCS); Van; curcumin	Carrier and antimicrobial	*S. aureus*-infected skin defect rats	The synergistic effect of broad-spectrum antibacterial Zn2+ and vancomycin	[199]
ZIF-8	Zn-MoS_2_	PS and antimicrobial	Skin wounds mouse	Photocatalytic; photothermal effects and the synergistic effect of zinc ions	[200]
ZIF-8	IGC; glucose oxidase (GOx); Fe^3+^; tannic acid (TA)	Carrier and PS	MRSA-induced abscess mice	Chemokinetic therapy and photothermal effects	[188]
ZIF-8	Mn^2+^/Mn^4+^	Antimicrobial	Full-thickness skin wounds in *S. aureus*-infected mice	Release of Zn^2+^	[201]
Zn-MOF	Chlorin (Ce6); alginic acid; poloxamer (F127)	Carrier and antimicrobial	*S. aureus*-infected skin defect rats	Destruction of bacterial membranes and ROS produced by PDT jointly exert an antibacterial effect	[202]
Cu-MOF	Gox; Carbon Dots (CDs)	Carrier	*S. aureus*-induced skin infection in diabetic male NIH mice	Cascade catalytic reaction converts endogenous glucose to radial hydroxyl radicals (•OH)	[203]
Ag-MOF	Bacterial cellulose (BC); Cur	Antimicrobial	The full-thickness skin defect rats	Release of Cur/Ag^+^	[204]
PCN-222 (Zr)	Bi-NPs	Antimicrobial	*S. aureus*-infected skin defect mice	Generation of ROS	[205]
PCN-224 (Zr)	. Dimethyloxalylglycine (DMOG); meropenem (MEM)	Carrier and PS	Full-thickness cutaneous wound mice	Synergistic chemo-photodynamic antimicrobial effect	[206]

**Figure 12:**
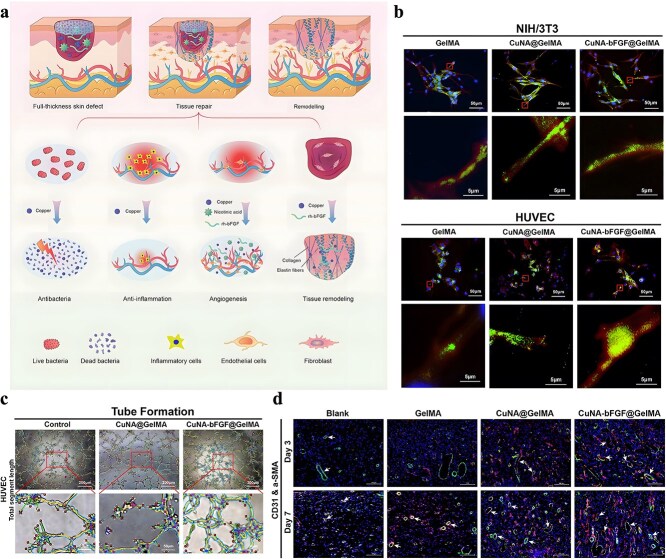
A kind of copper-based MOF material for infected skin wound. (**a**) Illustration of the multifunctional CuNA-bFGF@GelMA hydrogel for accelerating the process of wound repair [208] (reproduced from ref. [208]. Copyright 2021, BioMed Central). (**b**) Cell attachment and spreading behavior, migration, and tubule formation activities cellular merged fluorescent images of HUVEC and NIH/3T3 cells on GelMA, CuNA@GelMA, and CuNA-bFGF@GelMA hydrogels after 2 days postseeding [208] (reproduced from ref. [208]. Copyright 2021, BioMed Central). (**c**) The tube formation of HUVEC on matrigel after incubation with ionic extraction for 4 h [208] (reproduced from ref. [208]. Copyright 2021, BioMed Central). (**d**) Immunofluorescent staining of CD31 and α-SMA [208] (reproduced from ref. 208. Copyright 2021, BioMed central). *MOF* metal–organic frameworks, *CuNA* copper-nicotinic acid, *GelMA* gelatin methacrylate, ECM extracellular matrix, *bFGF* basic fibroblast growth factor, *NA* nicotinic acid

There are three main ways to use MOF for skin antibacterial; the first is pure MOF antibacterial. Based on the hydrothermal/pyrolysis strategy, a manganese-iron biatomic catalyst (Mn/Fe SACs) was developed for the modification of Mn/Fe@ZIF-8, which can be used for wound disinfection and abscess elimination. This versatile material has excellent stability and anti-inflammatory effects; in *in vitro* antimicrobial tests, it was found to be effective in catalyzing H_2_O_2_ production of ROS, thereby eliminating bacteria at the infected wound site. At the same time, the Balb/c mouse model of the wound treated with *S. aureus* infection using this material found that it can infiltrate into the infected wound to achieve satisfactory pathogen destruction, thereby eliminating the bacteria at the infected wound site, and, finally, the infected wound is completely healed, which is surprisingly Mn/Fe SACs have excellent biocompatibility and can highly promote the angiogenesis of new tissues in the wound area [198]. Similarly, they synthesized bamboo-like nitrogen-doped carbon nanotubes (N-CNTs@Co) coated with cobalt nanoparticles by pyrolysis of cobalt cyanide at high temperatures. It was found to have high antimicrobial efficiency in *in vivo* wound healing experiments [207]. We also mentioned earlier that the MOF as a carrier can improve the antibacterial efficiency of antibiotics, and studies have used this to use this for skin antibacterial. Vancomycin (VAN), for example, is a glycopeptide-based polymer antibiotic commonly used to treat *S. epidermidis* and MRSA. Lin *et al*. loaded Van onto MIL-53(Fe) to achieve a sustained release of Van with an antibacterial efficiency of 99.3% and no cytotoxicity [130]. Methods to further precisely control drug release and enable the targeted use of active substances at the right time and place. Song *et al*. used pH jumping reagent 2-nitrobenzaldehyde (*o*-NBA) to modify ZIF-8 and then loaded the antibiotic rifampicin (RFP) into the mesopores of *o*-NBA-modified ZIF-8 to construct a targeted antibiotic delivery system. The combination of pH-triggered precise antibiotic release and zinc ions enables the composite to significantly inhibit bacteria-induced wound infection and accelerate wound healing, indicating a switchable and synergistic antimicrobial effect [131]. The accumulation of acid under light irradiation ensures a controlled release of antibiotics and controlled degradation of ZIF-8, demonstrating the therapeutic potential of an MOF-based intelligent platform for controlling bacterial infections, enabling targeted use of antibiotics. Previous studies have found that the use of mild temperature photothermal therapy can promote angiogenesis; MOFs have good photothermal performance, and the combination of the two may not only achieve synergistic sterilization but also promote skin repair. In order to further accelerate the tissue formation of skin defects on the basis of antibacterial, Yang *et al*. prepared a copper-based MOF material (CuNA) with a doughnut-like structure. CuNA has a rough surface that contributes to the loading and release of basic fibroblast growth factor (bFGF), accelerating skin regeneration by promoting cell proliferation and angiogenesis. Then, it was mixed with GelMA to construct a multifunctional hydrogel CuNA@GelMA with excellent mechanical properties. (Figure 12a) Due to the synergistic effect of copper and bFGF, this hydrogel promotes cell migration and angiogenesis. (Figure 12b and c) As shown in Figure 12d, the resulting CuNA-bFGF@GelMA hydrogel significantly promoted the formation of new blood vessels and the deposition of collagen and elastin fibers in the full-thickness defect model of skin wounds, which greatly accelerated the wound healing process [208].

**Figure 13:**
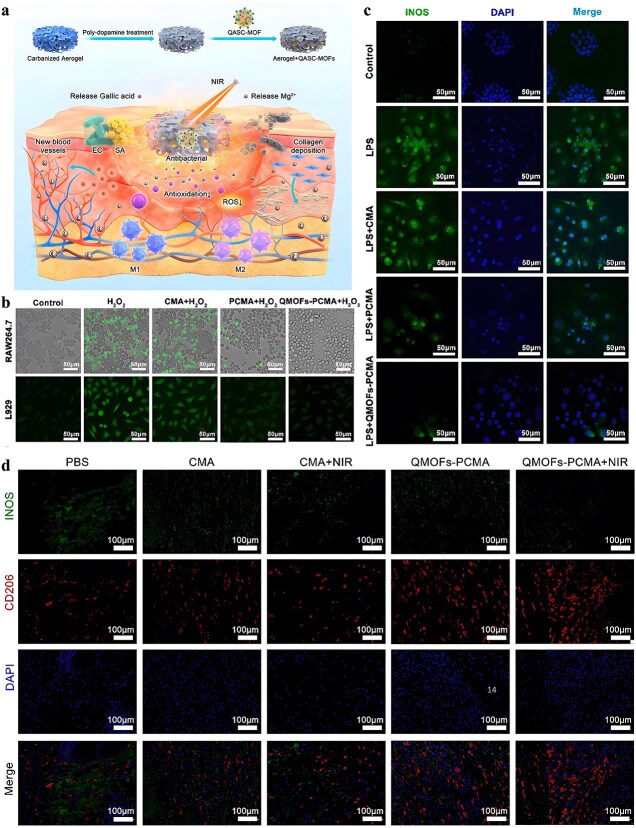
Bio-MOFs with photothermal therapy for eliminating biofilms. (**a**) The schematic diagram illustrates the preparation of gallic acid/magnesium^2+^ MOFs loaded carbonized mushroom aerogel and its application on biofilm-infected wound healing through photothermal therapy [209] (reproduced from ref. [209]. Copyright 2023, Elsevier). (**b**) The ROS deduction ability of CMA, PCMA, and QMOFs-PCMA on Raw264.7 and L929 cells. ROS was introduced by H_2_O_2_ for 0.5 h and stained with DCFH-DA [209] (reproduced from ref. [209]. Copyright 2023, Elsevier). (**c**) Fluorescent images show the effect of CMA, PCMA, and QMOFs-PCMA extracts on the polarization of Raw264.7 cells after 48 h of culture. The Raw264.7 cells were pretreated with LPS for 24 h to introduce an inflammatory status [209] (reproduced from ref. 209. Copyright 2023, Elsevier). (**d**) The effect of QMOFs-PCMA on the expression of macrophages in wound area [209]. (reproduced from ref. 209. Copyright 2023, Elsevier). MOFs, metal–organic frameworks. QMOFs-PCMA, magnesium/gallic acid bio-MOFs laden carbonized mushroom aerogel. *PTT* photothermal therapy, *CMA* carbonized mushroom aerogel, *IL-1β* interleukin-1β, *TNF-α* tumor necrosis factor-α, *QASC* quaternary amine salt functionalized chitosan

Previous research has shown that the application of mild photothermal therapy can stimulate the formation of new blood vessels; MOFs have excellent photothermal properties, and their combination can not only achieve enhanced sterilization but also support skin regeneration. Hyperelastic carbonized mushroom aerogels (CMAs) are biodegradable photothermal materials, and the development of a gel (QMOFs-PCMA) loaded on magnesium/gallic acid bio-MOFs has been described. This approach combines bio-MOFs with photothermal therapy to eliminate biofilms in skin wounds and modulate the immune microenvironment. (Figure 13a) Demonstrated the ability to clear ROS on H_2_O_2_-stimulated Raw264.7 and L929 cell models. (Figure 13b); At the same time, it showed excellent immunomodulatory ability in the Raw264.7 cell model (Figure 13c). *In vivo*, it can regulate the local immune response from a pro-inflammatory state to a proregenerative state, resulting in reduced expression of inflammatory cytokines and increased expression of anti-inflammatory cytokines [209].(Figure 13d).

#### Osteomyelitis

Osteomyelitis is typically caused by a bacterial infection and an inflammatory disease that affects the bone marrow, bone cortex, and periosteum. This condition involves acute inflammation, vascular damage, bone loss, and destruction, resulting in pain and harm to patients [210]. Traditional treatments, such as surgical debridement and antibiotic therapy, require long-term treatment and have a risk of recurrence [210, 211]. Effective treatment of osteomyelitis involves regenerating bone tissue while controlling infection and inflammation. In clinical practice, surgical debridement and systemic antibiotics are commonly used, but they may not ensure sufficient antibiotic concentration in the affected area. Fortunately, MOFs offer new and improved ways to address these challenges. MOF is more specific, efficient, and controllable in treating osteomyelitis.

**Figure 14:**
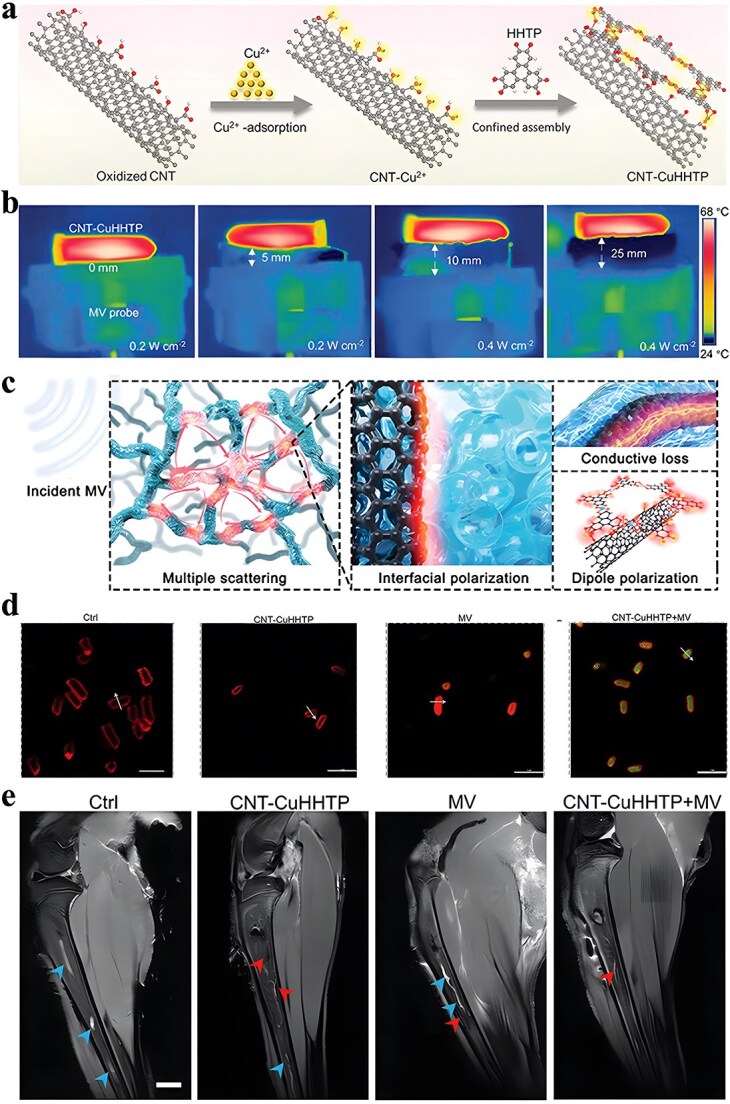
MOF-based materials used for osteomyelitis. (**a**) Schematic illustration for the synthesis of CNT-CuHHTP [212]. (reproduced from ref. [212]. Copyright 2023, Wiley Advanced). (**b**) Infrared thermal images of CNT-CuHHTP through different thicknesses of pork tissue under MV excitation [212] (reproduced from ref. 212. Copyright 2023, Wiley Advanced). (**c**) Microwave absorption mechanism of CNT-CuHHTP [212] (reproduced from ref. [212]. Copyright 2023, Wiley Advanced). (**d**) Fluorescent localization (above) of *E. coli* under different conditions [212] (reproduced from ref. 212. Copyright 2023, Wiley Advanced). (**e**) The MRI image results. The inflammation of the bacterial-infected bone marrow site is hyperintense (indicated by blue arrows) and accompanied by bone defects (indicated by red arrows) [212] (reproduced from ref. [212]. Copyright 2023, Wiley Advanced). *MV* microwave, *CNT* carbon nanotube, *MCT* microwaveocaloric therap, *MDT* microwave dynamic therapy, *ROS* reactive oxygen species, *HHTP* hexahydroxytriphenylene, *MRI* magnetic resonance imaging

The treatment of osteomyelitis using MOF often involves combining it with physical stimulation, such as ultrasound, to achieve a multimodal combined antibacterial effect for optimal efficacy. It has been reported that an interface-limited 2D MOF medium-voltage response system (CNT-CuHHTP) can be fabricated on carbon oxide nanotubes (CNTs), which has the synergistic effect of microwave and microwave kinetic therapy. As shown, the microwave thermal effect of CNT-CuHHTP can be used for in situ hyperthermia of tissues of different thicknesses, the material can disrupt *E. coli* cell membranes *in vitro*, and it is worth noting that the material has been shown to be effective in eradicating rabbit tibial osteomyelitis infected with *S. aureus* [212] (Figure 14). Similarly, Zeng *et al*. constructed a defective homoporphyrin-based MOF with enhanced sonocatalytic ability to treat osteomyelitis caused by MRSA. This MOF also exhibited good biocompatibility and antibacterial properties. It was able to change the immune microenvironment at the infection site and successfully prevent bone destruction [213].

In addition, the combination of NIR and MOF has been explored as an effective therapeutic measure. Teng *et al*. loaded iodine on the surface of ZIF-8 and found that under the action of NIR, ZIF-8 dissociates and releases iodine in a “triggered burst” manner. This generates reactive ROS in the liquid environment, resulting in an antibacterial effect. The iodine and its reaction products also improve the anti-infectious function through the synergistic effect of ROS production. The antibacterial efficacy of iodine-loaded ZIF-8-coated titanium implants was evaluated *in vivo* using a tibial intramedullary model of *S. aureus* infection. The results showed that the material effectively inhibited bacterial colonization and proliferation, leading to a significant reduction in the inflammatory response after treatment. This synergistic anti-infection strategy effectively addresses the challenge of iodine being difficult to dissolve while achieving both effective synergistic anti-infection function and simultaneous i*n vivo* bone formation ability [214].

#### Other infectious diseases

**Figure 15:**
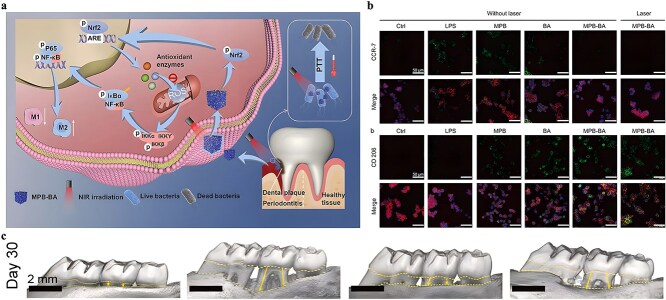
MOF-based materials for periodontitis. (**a**) Schematic illustration of antioxidant, anti-inflammatory mechanism, and antibacterial effects of MPB-BA in the treatment of periodontitis [215] (reproduced from ref. 215. Copyright 2022, Elsevier). (**b**) The effects of MPB-BA on macrophage polarization [215] (reproduced from ref. 215. Copyright 2022, Elsevier). (c) Micro-CT reconstructed images of the maxillary molar area [215] (reproduced from ref. [215]. Copyright 2022, Elsevier). *BA* baicalein, *MPB* mesoporous Prussian blue, *ROS* reactive oxygen species, *Nrf2* nuclear factor erythroid 2-related factor 2, *NF-κB* nuclear factor kappa-B

In addition to treating skin infections and osteomyelitis, MOFs have various applications in fighting against other diseases. There are multiple bacteria in the mouth that can lead to dental diseases. One of these diseases is periodontitis, which is caused by different oral pathogenic microorganisms including *F. nucleatus* and *S. aureus*. Traditional surgical interventions and antibiotic treatments are not very effective in treating periodontitis. Therefore, an MOF-based nanoplatform MPB-BA has been fabricated using mesoporous Prussian blue nanoparticles to support baicalein (BA) (Figure 15a). Under NIR irradiation, the nanomaterials exhibited highly effective photothermal antimicrobial activity. As Figure 15b shows, this nanomaterial enables macrophages to transition from the M1 phenotype to the M2 phenotype. This helps in the alleviation of inflammation (Figure 15c). Micro-CT results show that MPB-BA has a good *in vivo* therapeutic effect on periodontitis [215]. Long-term attachment of bacterial biofilms to tooth surfaces can lead to the development of tooth decay. To prevent dental caries and promote the natural healing of teeth, Li *et al*. conducted a hydrothermal synthesis of a Mg-MOF using gallic acid as an organic framework. They then coated the Mg-MOF with polydopamine (PDA) through a natural deposition process and facilitated the remineralization of calcium phosphate (CaP) on its surface using biomimetic methods. To make it more convenient for use, they designed a new foam mouth guard (MCP-CB) with easy operation, precise antibacterial properties, and an MOF-based structure. Under the low pH conditions of caries bacterial biofilms, SEM revealed the disintegration of the MCP polyhedral structure, leading to the release of antibacterial components such as Mg^2+^, Ca^2+^, and PDA, thereby reversing the caries-causing environment. *In vitro* experiments demonstrated that the MCP-CB exhibited excellent antibacterial properties, with the antibacterial rate reaching nearly 100% after near-infrared irradiation. Using a rodent caries model, the researchers observed that the MCP-CB effectively inhibited bacterial growth and disrupted biofilms. Furthermore, histological examination of the surrounding tissue showed good biocompatibility of the MCP-CB [216]. In contrast to the previous research on dental caries, Yu *et al*. utilized the beneficial properties of MOFs, such as their biocompatibility, pH responsiveness, and high loading performance, to enhance existing drugs used in clinical settings. They focused on improving the effectiveness of the anticaries vaccine called protein antigen c (PAc), which has the limitation of being relatively immunogenic and not being able to induce a low-level immune response. To address this issue, they incorporated ZIF-8 NP as an adjuvant to enhance the anticaries properties of PAc. The inclusion of ZIF-8 NP significantly increased the uptake of PAcs in lysosomes, leading to a robust immune response, inhibition of *Streptococcus mutans* colonization, and improved efficacy in preventing caries [217].

We mentioned earlier that ultrasound combined with MOFs can effectively treat osteomyelitis. In a similar vein, researchers have investigated the use of carbon@TiO_2_ nanoparticles derived from ZIF-8 in combination with ultrasound for the treatment of bacterial pneumonia. They evaluated the antibacterial effect of ZTNs through *in vitro* experiments using the plate counting method and found that the inhibition rate was above 80%. This indicates that ZTNs can effectively combat Gram-negative bacteria in a laboratory setting, offering a potential alternative to antibiotics for treating multidrug-resistant Gram-negative infections. By utilizing endotracheal atomized inoculation, ZTNs can be accurately delivered to the site of lung infection. In mouse models of lung infection, ZTNs, when exposed to ultrasound, were able to effectively eliminate *K. pneumoniae* in BALB/c, SCID, and NOD/SCID mice. Surprisingly, ZTNs achieved a 100% survival rate in NOD/SCID mice that were lethally infected. Combining MOFs and ultrasound-based sonodynamic therapy may prove to be an effective strategy against multidrug-resistant bacterial pneumonia [175].

Endophthalmitis, a common complication during the use of ophthalmic biomaterials and after ophthalmic surgery, is caused by pathogen infection. Here, in order to achieve effective PDT for bacterial infection and eradication of endophthalmitis biofilm, ZIF-8-PAA-MB@AgNPs@Van-PEG was constructed by modifying various antibacterial components such as final loaded ammonium benzene blue (MB), VAN, and silver nanoparticles with multiple modifications of ZIF-8. *In vitro* antibacterial studies were conducted against *E. coli*, *S. aureus*, and “methicillin-resistant” *S. aureus*. The results showed that the composite nanomaterial had significantly better antibacterial performance compared to any single strategy. *In vitro* experiments on retinal pigment epithelial cells demonstrated biocompatibility of the nanomaterial. *In vivo* experiments using mouse endophthalmitis models showed that the treatment had excellent antibacterial function and therapeutic performance. Various pathological examinations confirmed the effectiveness of the treatment. However, *in vitro* biocompatibility experiments have shown that high concentrations of these materials can lead to apoptosis of retinal pigment epithelial cells. However, the phototherapy system developed in this study offers a promising treatment option for the treatment of ophthalmic diseases [218].

### Challenge and future perspectives

**Figure 16:**
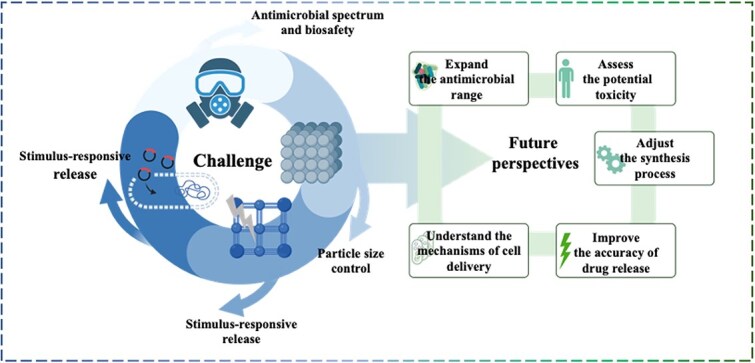
Challenge and future perspectives of MOFs. *MOFs* metal–organic framework

As the complexity of clinical bacterial infections increases, the threats to human life, health, and safety become more severe. Therefore, there is an urgent need to explore new antibacterial materials and methods. As mentioned earlier, MOFs can release metal ions and organic molecules to eliminate bacteria. They can also act as carriers for antibiotics, enhancing their antibacterial effect. Additionally, MOFs can be combined with ultrasound or near-infrared to achieve a broad-spectrum bactericidal effect, showing promising potential in biological applications. However, there are several challenges that MOFs face in practical applications, which currently limit their widespread use (Figure 16). Next, we will focus on its related challenges.

#### Antimicrobial spectrum and biosafety of metal–organic frameworks

Indeed, MOFs have excellent antibacterial properties. However, current research on the antibacterial properties of pure MOFs is mainly focused on bacteria such as *S. aureus*, *E. coli*, and *P. aeruginosa*. The antibacterial range is relatively limited, and the antibacterial effects are also limited. The concern is that after prolonged antimicrobial treatment with high concentrations of metal ions, bacteria will develop resistance to these metal ions. The combination of MOFs with other therapies such as phototherapy and sonotherapy can enhance the antibacterial ability of MOFs. As mentioned earlier, the multiple modifications of ZIF-8 for the treatment of intraocular inflammation have shown that this composite nanomaterial has significantly better antibacterial properties than any single strategy and also exhibits excellent antibacterial effects against drug-resistant bacteria. However, the range of bacterial species involved in current research on the antibacterial properties of MOFs still needs to be expanded.

To further explore the biomedical applications of MOFs, it is crucial to consider the potential toxicity of MOFs and conduct a thorough evaluation. Due to the diversity of MOFs in structure and species, as well as the complexity of the internal environment of organisms, the toxicity of MOFs is related to a variety of factors, including composition, morphology, size, and stability. However, these metal ions and organic substances can also cause damage to organs and potentially disrupt the immune system to some extent. For instance, the multifunctional nanomaterials modified using ZIF-8 mentioned earlier can effectively treat endophthalmitis through antibacterial actions. However, *in vitro* biocompatibility experiments have revealed that high concentrations of these materials can lead to apoptosis of retinal pigment epithelial cells [218]. A potential strategy to mitigate MOF toxicity is to utilize endogenous metal ions and biocompatible organic ligands. Although certain MOF-based materials have shown success in short-term *in vivo* experiments with animals, concerns remain about their potential long-term effects, especially at higher concentrations or with prolonged exposure. Biodegradability is another key aspect to consider [219]. When considering future biomedical applications, it is important to evaluate the long-term toxicity and metabolic mechanism of MOF degradation. A comprehensive assessment of the potential toxicity of MOFs, as well as understanding the key factors that influence toxicity, are crucial for advancing future clinical research on MOFs. In addition, combining MOFs with existing therapeutics or drug treatments requires comprehensive planning and coordination within current healthcare practices [219].

#### Particle size control of metal–organic frameworks

The safety of MOFs is crucial in determining whether they can be used in clinical research. Then, the size of the MOF particles is one of the key factors to consider in order to ensure that they are nontoxic for biomedical applications [220, 221]. Reducing the size of MOFs to the nanoscale has been found to enhance their pharmacological properties, such as drug delivery efficiency and controlled release [222]. Nano-MOFs appear to be more biocompatible compared to larger micron-sized MOFs. However, it is important to note that when the particle size is smaller than ~200 nm, the toxicity increases as the size decreases [223]. However, once the particle size reaches a specific threshold, MOFs become highly unstable and prone to clustering and settling [224, 225]. Additionally, when the particle size of the MOF is too small, the small pore size limits the access of larger molecules and significantly impacts their loading capacity [226, 227]. The particle size of MOFs significantly impacts their use in the biomedical field. Firstly, reducing the particle size increases its specific surface volume, leading to increased reactivity. Secondly, smaller particles have greater penetration ability. The mechanism of endocytosis depends on the particle size [228]. The size of MOFs plays a crucial role in their uptake by cells and subsequent transport within biological systems. An appropriate particle size can facilitate their penetration of biological barriers, accumulation at specific sites, and passage through the cell membrane. According to Wang *et al*. [229], MOFs with a particle size of 90 nm exhibit the highest efficiency in being endocytosed by living cells and regulating their release from endosomes into the cytoplasm, so precise control and optimization of the particle size is necessary for MOF materials used in biomedical applications.

Studies have shown that the particle size of MOFs can be controlled by adjusting reaction parameters (such as temperature and pH) or introducing auxiliary additives (such as coordination regulators or surfactants) [230]. Wang *et al*. have successfully demonstrated the ability to control the size of MOF crystals by adjusting the amount of metal nodes added during the seed generation process [231]. This method allows for precise and controllable regulation of the particle size of the material. Additionally, this method has shown promising results in effectively regulating the size of ZIF-8 and composite NPs@ZIF-8 materials, making it a versatile approach. In the future, our aim is to develop even simpler and more effective methods for precisely controlling the particle size of MOF materials, with the goal of advancing their applications in biomedicine.

#### Stimulus-responsive release of metal–organic frameworks

In the previous description, it is not difficult to find that while MOFs exhibit effective antibacterial effects in the experimental phase, the antibacterial performance of MOF matrix composites and stimulus-responsive MOFs (such as those responsive to pH, photothermal, and ultrasound) is significantly higher compared to simple MOFs. When the drug is loaded by MOF or directly synthesized as an organic ligand to prepare MOF matrix composites, the antibacterial effect of MOFs is enhanced, and the antibacterial mechanism of individual components can be coordinated, so MOF matrix composites have better antibacterial effects. At the same time, high-dose and long-term use of metal ions can lead to drug resistance, so the design and development of MOF materials that can respond to multiple stimuli and respond quickly and efficiently is the main direction of future research. Additionally, modifying MOFs with stimuli-responsive or targeted substances enables precise controlled release of drugs at specific times and locations. For example, US combined with MOFs can be employed to treat deep tissue infections, increasing bioavailability and antimicrobial properties. However, some targeted and stimulus-responsive materials may be harmful to organisms, limiting their clinical application. Given the growing need for MOFs in the field of nanomedicine, it is crucial to conduct thorough and systematic research on the processes of drug loading, release, and degradation of MOF-based drug delivery systems [232, 233]. Additionally, it is imperative to address the issues of chronic and acute toxicity associated with stimulus-responsive MOFs. Moreover, conducting pharmacokinetic studies on the absorption, distribution, metabolism, and excretion of MOFs *in vivo* is critical for advancing their clinical development. However, there is currently no available information on the pharmacokinetics of MOFs. Additionally, it is imperative to address the issues of chronic and acute toxicity associated with stimulus-responsive MOFs.

Currently, the focus of research on stimulus-responsive MOFs is mainly on their application in treating tumors/cancers, with limited studies on their antibacterial properties. It is necessary to broaden the scope of applications for stimulus-responsive MOFs. To expand the use of MOFs in antibacterial treatments, it is important to consider the design of MOF-based oral delivery [234]. Administering MOFs orally may be the preferred method for treating deep-seated antimicrobial diseases. As the use of stimulus-responsive MOFs in biomedical applications continues to grow, it is crucial to further enhance the accuracy, quantification, and control of drug release in order to meet diverse requirements.

#### Intracellular antimicrobial of metal–organic frameworks

The hydrophilicity of commonly used antibiotics greatly limits their ability to pass through membrane barriers, accumulate within infected cells and thus kill intracellular bacteria. This can also lead to low treatment efficacy and the emergence of antibiotic resistance. In our previous discussion of the antibacterial mechanism of MOFs, we mentioned that positively charged metal ions in MOFs can adhere to negatively charged bacterial membranes through electrostatic interactions, thereby disrupting the bacterial membrane, releasing ROS, and affecting bacterial protein function, ultimately resulting in antibacterial effects. However, relying solely on charge adsorption is not sufficient to increase drug accumulation within infected cells. We should utilize the unique properties of MOFs, such as their surface rich in functional groups, to modify them by targeting moieties or hydrophilic polymers [235], thereby imparting them with stealth characteristics. For instance, It is known that folic acid (FA) is a vital nutrient necessary for bacterial nucleotide production. Consequently, we can attach FA to the surface of an MOF, which will greatly enhance the absorption of the MOF by bacteria. Chowdhuri *et al*. took advantage of this and incorporated FA into the Van-loaded ZIF-8 system to enhance the effectiveness of the antibacterial properties against MRSA. Due to the abundance of FA receptors on the bacterial membrane, FA-modified Van@ZIF-8 can easily enter *S. aureus* through a process called endocytosis. This approach enables targeted and specific release of Van within the bacterial cell [141]. Enhancing the ability of MOFs to accumulate within pathogen cells provides an excellent opportunity for eradicating persistent infections based on MOF-based antibacterial strategies. Although significant progress has been made in the antimicrobial properties of MOFs, the development of therapeutic platforms using multifunctional MOFs is still in the early stages of research. As mentioned before, MOF nanoparticles have the ability to reach intracellular bacteria that are protected. One of the main challenges in using MOFs in clinical applications is the limited understanding of how they interact with cells and penetrate them. However, the effectiveness of MOF accumulation in the bacterial compartment of cells is still unclear. As mentioned above, the study of FA-modified Van@ZIF-8 did not involve animal experiments, and even cell experiments did not carry out proliferation experiments such as CCK8 to prove that it has good biocompatibility [141]. Understanding the cellular uptake and transport of MOFs is a complex process that involves selecting appropriate biological models and considering the physicochemical properties of the particles. It is also important to expand cell-level assessments to more complex cellular models, such as organoids [236]. To assess the adequacy of bacterial targeting and treatment efficacy, multiple studies using different *in vivo* models are needed [237]. To successfully translate MOFs into clinical applications, understanding the cellular delivery and uptake of MOFs remains one of the most critical issues in the field.

#### Different applications of metal–organic frameworks

Due to the special physical and chemical properties of MOFs, they show great potential in antibacterial applications; the antibacterial activity of MOFs can be divided into their intrinsic antibacterial activity and their function as drug carriers;

The presence of metal ions makes MOFs have intrinsic antibacterial activity. As mentioned above, metal ions can destroy bacterial cell membranes, generate ROS, and inhibit DNA and protein synthesis after release; through a series of molecular damage mechanisms, the normal biochemical reactions and metabolic active cells of microorganisms are blocked, eventually leading to bacterial death. In addition, positively charged metal ions also help them adsorb to the surface of bacteria and interfere with their normal metabolic processes. The different antibacterial mechanisms of MOFs lead to the diversification of their antibacterial effects, with broad-spectrum antibacterial effects, while effectively avoiding drug resistance. In short, MOFs have become a broad-spectrum antibacterial material suitable for surface coatings of medical devices.

Due to the high specific surface area and porous structure of MOFs, the application of MOFs as drug carriers in the field of antibacterial has also attracted much attention. MOFs can be used as carriers of antibacterial drugs such as antibiotics, thereby enhancing the antibacterial properties of loaded drugs and enhancing the stability and utilization of antibacterial drugs. The combination of inorganic antimicrobial agents and MOFs exerts a synergistic effect to maximize the antibacterial effect. In addition, MOFs can not only act as carriers but also respond to specific stimuli in the local microenvironment through surface functionalization to achieve controlled release and therapeutic effects. Such drug-loaded MOFs are mainly used in the precise treatment of *in vivo* infections, the regulation of disease microenvironment, and the application of long-term antibacterial diseases.

In short, the intrinsic antibacterial activity of MOFs and their drug carrier function are both independent and have their own advantages. The two show their antibacterial ability from direct and indirect levels, respectively; the former is simple to synthesize, emphasizes the function of the material itself, and is suitable for drug-resistant bacteria and broad-spectrum antibacterial; the latter focuses on the precise delivery of drugs and the personalized needs of treatment. According to the different needs of actual application scenarios, the intrinsic antibacterial activity of MOFs and their function as drug carriers can provide a variety of options for the development of antibacterial materials.

## Conclusions

In recent years, materials based on MOFs have gained widespread attention in various antibacterial applications due to their high surface area, controlled/stimuli-responsive release of antibacterial components, adjustable size, and sustained release of metal ions. We summarized the latest advances in the use of MOFs as antibacterial agents, focusing on their preparation methods, basic antibacterial mechanisms, antibacterial performance of pure MOFs, antibacterial applications as carriers, and applications in infection-related diseases. Although MOFs still face long-term challenges in biomedical applications, such as precise control of particle size and further improvement in biocompatibility, the potential of MOFs in antibacterial applications is enormous. It is believed that with further research and development, novel MOFs with improved biocompatibility and targeting capabilities can offer new therapeutic strategies for various diseases caused by bacterial infections.

## Data Availability

Not applicable.
